# Molecular Dynamics Simulations of Cellulose and Dialcohol Cellulose under Dry and Moist Conditions

**DOI:** 10.1021/acs.biomac.3c00156

**Published:** 2023-05-11

**Authors:** Patric Elf, Hüsamettin Deniz Özeren, Per A. Larsson, Anette Larsson, Lars Wågberg, Robin Nilsson, Poppy Thanaporn Chaiyupatham, Mikael S. Hedenqvist, Fritjof Nilsson

**Affiliations:** † School of Engineering Sciences in Chemistry, Biotechnology and Health, Fibre and Polymer Technology, KTH Royal Institute of Technology, SE-100 44 Stockholm, Sweden; ‡ Department of Chemistry and Chemical Engineering, Chalmers University of Technology, SE-412 96 Gothenburg, Sweden; § FibRe Centre for Lignocellulose-based Thermoplastics, KTH Royal Institute of Technology, Stockholm, SE-100 44, Sweden; ∥ FibRe Centre for Lignocellulose-based Thermoplastics, Chalmers University of Technology, SE-412 96 Gothenburg, Sweden; ⊥ FSCN research centre, Mid Sweden University, 85170 Sundsvall, Sweden; # Division of Glycoscience, Department of Chemistry, KTH Royal Institute of Technology, AlbaNova University Centre, SE-106 91 Stockholm, Sweden

## Abstract

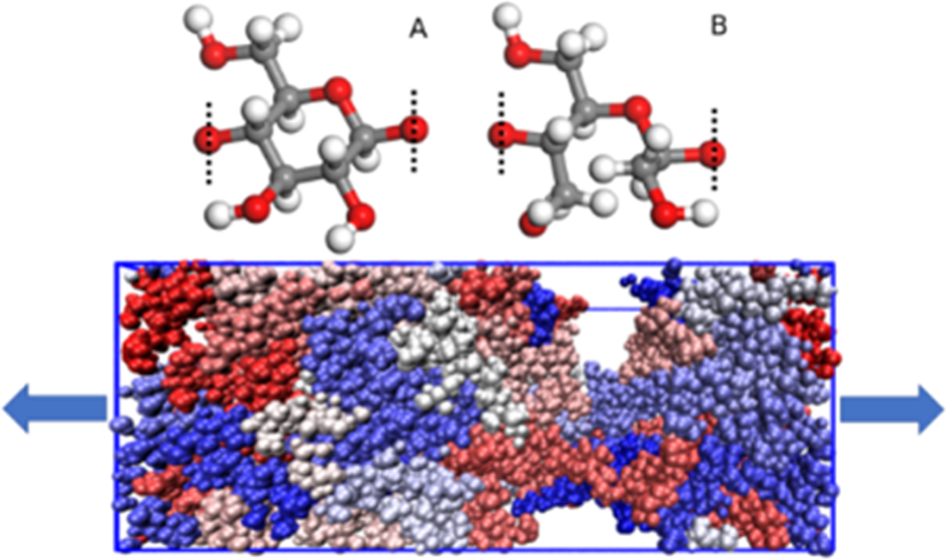

The development of wood-based thermoplastic polymers that can replace synthetic plastics is of high environmental importance, and previous studies have indicated that cellulose-rich fiber containing dialcohol cellulose (ring-opened cellulose) is a very promising candidate material. In this study, molecular dynamics simulations, complemented with experiments, were used to investigate how and why the degree of ring opening influences the properties of dialcohol cellulose, and how temperature and presence of water affect the material properties. Mechanical tensile properties, diffusion/mobility-related properties, densities, glass-transition temperatures, potential energies, hydrogen bonds, and free volumes were simulated for amorphous cellulosic materials with 0–100% ring opening, at ambient and high (150 °C) temperatures, with and without water. The simulations showed that the impact of ring openings, with respect to providing molecular mobility, was higher at high temperatures. This was also observed experimentally. Hence, the ring opening had the strongest beneficial effect on “processability” (reduced stiffness and strength) above the glass-transition temperature and in wet conditions. It also had the effect of lowering the glass-transition temperature. The results here showed that molecular dynamics is a valuable tool in the development of wood-based materials with optimal thermoplastic properties.

## 1 Introduction

Thermoplastic materials from renewable resources are rapidly attracting more attention as sustainability becomes increasingly important in the society. Wood-derived materials are particularly interesting, due to the natural abundance, biodegradability, and regrowth of wood. All three main components of wood (cellulose, hemicellulose, and lignin) are interesting when developing new bio-based thermoplastics; however, in this study, the focus is on materials originating from cellulose, a resource of high interest for the forest industry and society today.^1,2^

The thermoplastic properties of cellulose-based materials are depending on a multitude of factors, including molecular structure, intermolecular interactions, crystallinity, fibril structure, and the hierarchal structure of fibers as well as the presence of plasticizers and chemical modifications. To facilitate the replacement of fossil-based plastic materials with bio-based cellulose materials, fundamental knowledge about the underlying mechanisms that influence the processing and final properties is required.

Dialcohol cellulose, i.e., modified cellulose where the bond between the *C*_2_ and *C*_3_ carbon atoms in the ring structure is cleaved, shows interesting properties since this modification has led to increased ductility and decreased glass-transition temperature (*T*_g_).^3,4^ This indicates that several thermo-mechanical properties of cellulose materials can be improved by ring opening of the glucose unit. One important goal of this study is to examine how and why the degree of ring opening in cellulose influences the thermoplastic properties of the cellulose material. Atomistic molecular dynamics (MD) computer models of systems with disordered amorphous cellulose were used to assess the effects of ring opening on the molecular behavior of the cellulose, not considering the separate complex effects of changing the fiber morphology and the supramolecular structure of the cellulose in the fiber wall.

When replacing a fossil-based plastic material, the new bio-based material should for economic and practical reasons have approximately the same characteristics as the former material. Ideally, the new material should also be processable in existing processing equipment, to avoid having to develop new less effective processing techniques, the former that have been optimized over many years.

For polymeric materials, such as cellulose derivatives, the thermoplastic properties depend on polymer chain interactions, including hydrogen bonds, dispersive and electrostatic interactions, and chain entanglements.^3,5,6^ In the case of cellulose, the presence of water will also affect the thermoplastic and mechanical properties; it is well known that its plasticizing effect will have an impact on, e.g., *T*_g_.^7^

Experimental methods, requiring preparation and characterization of physical samples, are typically both time-consuming and labor-intensive. This limits the maximum number of samples and thus the number of variables that can be studied. Computer simulations do not have this limitation and can be used as a valuable complement. Molecular dynamics simulation is a useful technique for investigating atomistic interactions in cellulosic and polymeric materials, both in crystalline phases and amorphous systems, since it is relatively fast and often reproduces material property trends accurately.^1,2,5,8,9^

MD simulations are efficient for predicting and explaining molecular interactions and material properties but are still naturally limited mainly by the available computational resources, which constrain the number of atoms and the time scale for the simulations. Current time limits are in the nano- to microsecond range, which, however, is sufficient for describing several material properties, such as density or *T*_g_. The limitations of MD can partly be mitigated with periodic boundary conditions, mimicking infinitely large molecular systems.

When modeling polymers with MD, the chains are shorter than most real polymer chains, to enable system equilibration within reasonable CPU time, but still sufficiently long to avoid spurious contributions from chain ends, particularly in low-mobility systems.^5^ MD simulations involving time-dependent processes, such as tensile testing, are by necessity performed at very high deformation rates, leading, e.g., to higher tensile strengths and modulus than measured experimentally.^10^ Awareness of the differences between experimental and simulated polymer systems is thus necessary to correctly interpret the results. Simulations have a high potential to accelerate future material research, but experimental verifications of key findings are still necessary.

In this work, fully atomistic MD simulations were performed for amorphous cellulose and dialcohol cellulose systems. The influence of temperature, water content, and degree of ring opening (i.e., degree of conversion to dialcohol cellulose) was investigated. Two temperatures (room temperature (23 °C) and 150 °C, a temperature that has been used to extrude cellulosic materials^11^) and two water contents (0 and 25 wt %) were used. The water content was chosen to span over the actual water content used for the previously extruded material.^12,13^ The degree of modification, i.e., the percentage of cellulose repeat units being converted into ring-opened (dialcohol) cellulose, was evenly distributed between 0 and 100% conversion (0, 25, 50, 75, and 100%). To examine how the three variables influenced the material, several material properties, including pressure–volume–temperature (PVT), free volume, structural changes, mobility/diffusivity, tensile properties, and electrostatic interactions (e.g., hydrogen bonds), were investigated. Since it is difficult to fully encapsulate the complexity of real cellulosic materials, the molecular systems in this study were simplified to avoid higher hierarchical structures, such as fibril structures, and focus on amorphous systems, which can still indicate the behavior of the materials on a grander scale. The reason for comparing MD with experiments despite the differences in microstructure/crystallinity is to see the general trends in, e.g., how the ring opening affects the glass-transition temperature. The MD results were, when possible, compared to the properties of real 100% ring-opened dialcohol cellulose (samples prepared for this study), but the mechanical properties were compared to DMA measurements of dialcohol cellulose with 0–40% ring openings (samples from a previous study).^14^

## 2 Method

### 2.1 Molecular Dynamics Simulations

#### 2.1.1 System Descriptions

**Figure 1:**
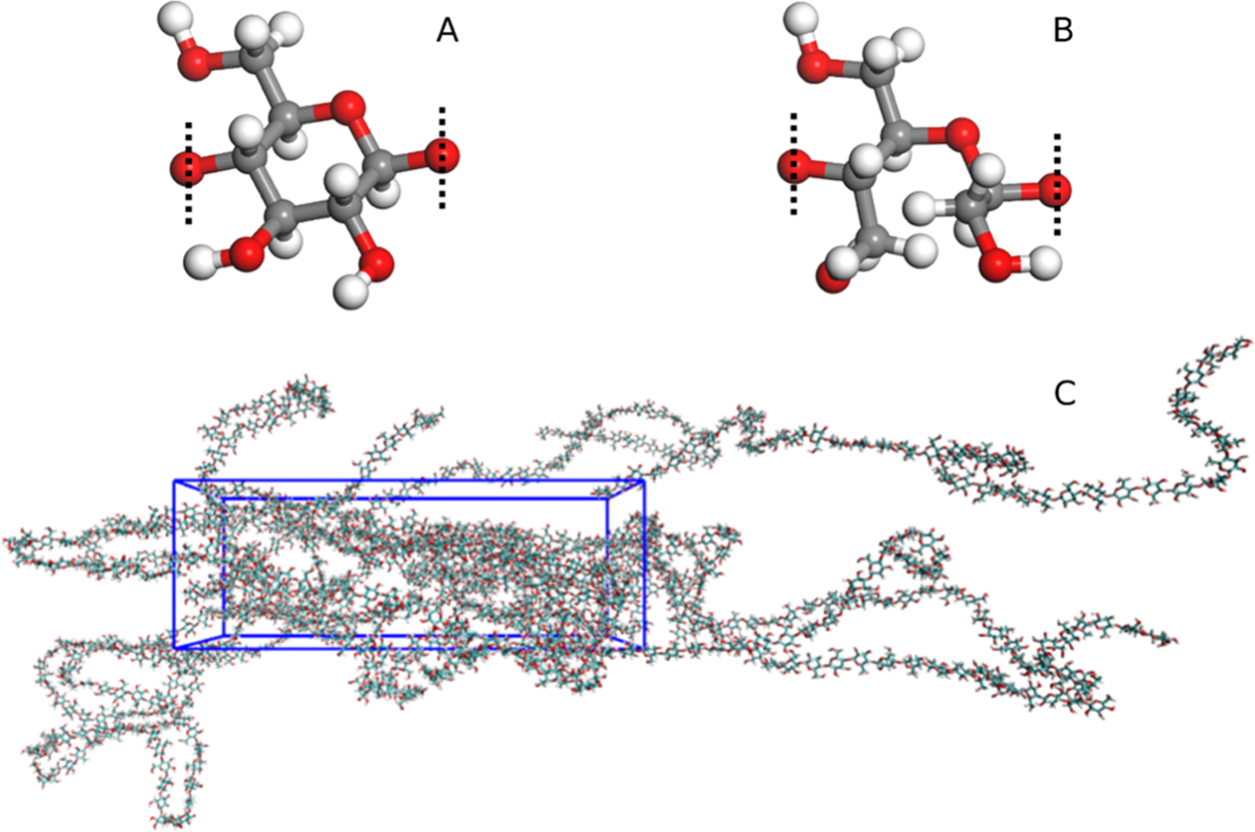
Molecular structures. (A) A repeating unit of cellulose, (B) a repeating unit of dialcohol cellulose monomer, and (C) a representative computational box of an MD system after 100% stretching. Dotted lines show which oxygen atom binds to the neighboring monomer.

Cellulose and dialcohol cellulose repeating units were prepared in Biovia Materials Studio (2016). Cellulose was constructed by using d-glucose units, connected between the *C*_1_ and *C*_4_ carbons using β-glycosidic bonds. Dialcohol cellulose was then created by starting from a cellulose template, where the bond between the *C*_2_ and *C*_3_ carbons was removed, and the resulting structure was hydrogenized. The dry polymer systems contained 20 chains, whereas the wet systems, which contained 25 wt % water, had 16 chains. Each chain comprised 50 repeating units, resulting in structures with ca. 20,000 atoms. The number of chains was chosen such that all systems would have approximately the same number of atoms, be sufficiently large, and be reasonably fast to simulate, i.e., not contain too many atoms. Pure cellulose and dialcohol cellulose chains were created using a single type of repeat unit, whereas mixed systems with 25, 50, and 75% ring openings were created using a script that generated chains with a certain fraction of ring openings, and randomly placed cellulose and dialcohol cellulose units. Another script was used to convert the Material Studio data files to GROMACS-compatible format. A 21-step decompression method was used to equilibrate the system,^5^ after which a 10 ns NPT simulation was used to set the system to the desired simulation temperature. The Debyer software (https://github.com/wojdyr/de-byer) was used to obtain the X-ray diffraction (XRD) patterns of the simulated systems, using 1/3 of the box length as cutoff, a step size of 0.1 Å, and a wavelength of 1.54 Å. Repeating units of cellulose and dialcohol cellulose are shown in Figure 1a,b respectively, together with a representative simulation box after 100% stretching in Figure 1c, meaning the box has been extended to twice its initial length. The force-field parameters are given in Tables S1 and S2.

#### 2.1.2 Density, Glass-Transition Temperature, and Thermal Expansion

To predict the density, specific volume, glass-transition temperature (*T*_g_), and coefficient of thermal expansion (CTE) of the materials, a series of isothermal–isobaric ensembles (NPT) simulations using Parrinello–Rahman pressure coupling were performed at 1 atm pressure. The systems, with and without water, started at 575 K (302 °C) and 800 K (527 °C), respectively, going down to 150 K (−123 °C) in decrements of 25 K. The reason for the lower starting temperature for the systems with water is because the wet systems become unstable around 600 K, due to boiling of the water model. Experimentally, cellulose would degrade at lower temperatures than 600 K, but as the model doesn’t allow bond breaking or degradation, the simulated temperatures are feasible. The molecular systems were equilibrated for 30 ns at the starting temperature, using a time step of 1 fs. Due to the small temperature decrements, a somewhat shorter equilibration time (10 ns) was used for the subsequent temperature steps. The specific volume at each temperature was calculated as the average volume of the simulation box during the final 0.5 ns of the simulation using GROMACS built-in function, gmx energy. The system densities at 296 K (23 °C) and 423 K (150 °C) were evaluated in a similar fashion, using gmx energy, for the final 0.5 ns of 10 ns equilibrations at these temperatures.

The precision of the generated PVT data was assessed using three tests: (i) Triplicate samples were evaluated for representative systems, (ii) two water models (TIP3P and TIP4P) were compared, and (iii) cooling and heating PVT data were compared.

*T*_g_ was calculated using broken stick regression, where two straight lines were fitted to specific volume data at two different temperature regions. One line was fitted in the glassy phase below *T*_g_ and one in the rubbery phase above *T*_g_. The intersection between the two lines was defined as *T*_g_. A linear fit using the seven lowest and highest temperatures was used.

The coefficient of thermal expansion (CTE) was determined as the reciprocal specific volume of the material multiplied by the volume change with respect to temperature
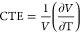
1The derivative, which typically increases with temperature, corresponds to the slope of the specific volume vs temperature curve. Here, the slope in the temperature range 150–225 K was used for CTE, i.e., the slope of the fitted line below *T*_g_ in the broken stick regression.

#### 2.1.3 Tensile Properties

Deformation simulations were performed using a semi-isotropic Parinello–Rahman pressure coupling. The systems were isotropically coupled in two of the directions and were deformed in the third direction at a rate of 0.001 μm/ns for approximately 6 ns, or until the system had reached 100% strain. Note that the chain lengths and the system size can affect the yield strength significantly^5,15,16^ and that large systems are recommended. The (true) stress σ was defined as the negative pressure tensor in the deformation direction:
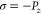
2The pressure tensor in the *z*-direction (*P*_*z*_) fluctuates significantly, and to compensate for this, the stress was calculated as the rolling average over strains ±2.5% from the current strain. To account for the absence of measurement points before the start of the simulation, the stress was set to zero at these points. The yield strength was determined as the maximum value in the averaged curve between 3 and 97% strain. The engineering strain ϵ was used
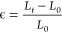
3where *L*_*t*_ is the length of the simulation box (in the direction of extension) at time *t* and *L*_0_ is the initial length. The Young’s modulus, *E*, was defined as the slope of the initial linear part of the stress–strain curve
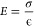
4To account for possible numerical artifacts during the start of the deformation, Young’s modulus was calculated between 0.3 and 3% strain. The tensile simulation box was done with extension for the initial systems in *X*, *Y*, and *Z* directions as is standard practice^15^ to make sure, by rotating the simulation box, that we have similar tensile responses in all directions.

#### 2.1.4 Diffusion and Mobility

The mobility of water molecules and polymer chains was determined from their three-dimensional Brownian motion. The diffusivity *D_X_* of species *X* (water or polymer chain) was computed from the mean square displacement (MSD) of 10 ns canonical ensembles (NVT) simulations, using Einstein’s relation^17^
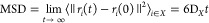
5where *r*_*i*_(*t*) is the center of mass of molecule *i* at time *t*. Only the linear or near-linear part of the MSD curves was used, to avoid artifacts from the initial ballistic behavior, subsequent cage-like diffusion, and from the poor statistics at the end of the curve.

The diffusivity/mobility of a species is coupled partly to the free volume of the system, i.e., the unoccupied space in the system, which is related to the molecular packing efficiency. The free volume fluctuates slightly due to molecular movement and oscillations, and it is affected by the molecule structure and intermolecular forces. If the molecular cohesion is high, the molecules become more tightly packed, and the free volume decreases.^18^ For penetrant diffusion, the effective free volume depends on the size of the penetrant molecules. The fractional free volume (FFV) is defined as:
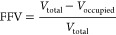
6where *V*_occupied_ is the volume occupied by the van der Waals volume of the atoms and *V*_total_ is the total volume in the system. By inserting spherical probes with different radii in the molecular system, the FFV and the FFV distribution were determined as a function of probe radius. The FFV can be used to predict the diffusivity of penetrant molecules in the system.^19^

#### 2.1.5 Hydrogen Bonds

Hydrogen bonds are common in polar hygroscopic polymers and play an important role in the molecular mobility of the polymer and its interactions with water. In the simulations, hydrogen bonds were defined as configurations with donor-acceptor distances <0.35 nm and hydrogen-donor-acceptor angles <30°. The hydrogen-bond time autocorrelation function *C*_HB_(*t*) was computed as:
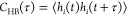
7where τ is a specific time period and *h*_*i*_(t) is a binary function, which is 1 if hydrogen bond *i* exists at time *t* and is 0 otherwise. Hydrogen-bond interactions were computed over a 10 ns interval for each combination of species, e.g., polymer–polymer, polymer–water, and water–water. As the polymer systems were quite immobile and a significant portion of the hydrogen bonds had a much longer lifetime than the simulation time of 10 ns, *C*(τ) was fitted to an exponential decay function where term *i* with weight *K*_i_ corresponded to process *i*:
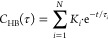
8
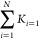
9Since *N* = 1 or 2 is usually sufficient for rapid processes like water–water interactions,^20^*N* = 2 was used when the data was extrapolated to 100 ns. The hydrogen-bond density was computed as the average number of hydrogen bonds over the 10 ns simulation, divided by the volume of the computational box. Integrating *C*_HB_ over time, using trapezoidal numerical integration, gives an estimate of the average hydrogen-bond lifetime τ_HB_:
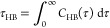
10

### 2.2 Experimental Section

#### 2.2.1 Sample Preparation

First, a dialdehyde cellulose solution was prepared, which in turn was converted to dialcohol cellulose. A sodium metaperiodate solution (Sigma-Aldrich, Schnelldorf, Germany) and microcrystalline cellulose, MCC, (Avicel PH-101, FMC BioPolymers Newark, Delaware) was mixed at a molar ratio of 1.1 sodium metaperiodate/1,4-anhydro-d-glucose units by dissolving the sodium metaperiodate in 1500 mL of deionized water in a 2 L Erlenmeyer flask. Isopropanol (Sigma-Aldrich, Schnelldorf, Germany) was then added as a scavenger, after which the pH was adjusted to 3.6–4 using acetic acid. An amount of 30 g of MCC, which had been dried in an oven at 50 °C, was then added to the solution and stirred at 200 rpm at room temperature. The solution was kept in a dark environment until the consumption of sodium metaperiodate, determined by UV–vis spectroscopy at 290 nm (Cary 60 UV/vis, Agilent Technologies), corresponded to a degree of modification of 100%. The solution was then washed using distilled (DI) water repeatedly until the UV absorbance of the washing water was similar to that of the DI water used. This wet dialdehyde cellulose was then stored at 4 °C until further use. To prepare dialcohol cellulose, the dialdehyde cellulose was resuspended in 200 mL of DI water in a 2 L flask for at least 30 min, after which 0.02 M monobasic sodium phosphate (Sigma-Aldrich, Schnelldorf, Germany) was added. A mass of 15 g of sodium borohydride (Sigma-Aldrich, Schnelldorf, Germany) and 100 mL of DI water were then slowly added dropwise into the dialcohol suspension. The suspension was stirred at 200 rpm for 4 h at room temperature, after which it was dialyzed for 1 week against DI water, and then dried in an oven at 40 °C.

#### 2.2.2 Measurements

##### 2.2.2.1 Thermogravimetry

Thermogravimetry analysis (TGA) measurement, using a Mettler Toledo TGA/DSC 1, was performed with a 5 mg sample placed in a 70 μL alumina crucible. It was heated from 30 to 600 °C with a heating rate of 10 °C/min using a N_2_ purge gas flow of 50 mL/min.

##### 2.2.2.2 Differential Calorimetry

Differential scanning calorimetry (DSC) measurements were performed using a Mettler Toledo DSC 1. The sample with a weight of 5.5 mg was placed in a 40 μL aluminum pan having a pierced lid. The temperature was first kept at −30 °C for 5 min, whereafter it was raised to 220 °C at a heating rate of 20 °C/min. After 5 min at 220 °C, the temperature was decreased to −30 °C at a cooling rate of 20 °C/min, and kept at −30 °C for 5 min, before the whole cycle was repeated. The N_2_ purge gas flow was 50 mL/min. The high heating and cooling rates were chosen to be able to observe the glass transition more clearly.

##### 2.2.2.3 Fourier Transform Infrared Spectroscopy

Fourier transform infrared (FTIR) spectroscopy absorbance was measured using a PerkinElmer Spectrum 100 FTIR Spectrometer from 600 to 4000 cm^–1^ with a built-in universal ATR. The scanning step was set to 1 cm^–1^ and with a resolution of 4 cm^–1^. 16 scans were recorded for each spectrum.

##### 2.2.2.4 X-ray Diffraction

A PANalytical X’Pert Pro was used for the XRD measurements, using a Cu Kα radiation source (wavelength of 1.54 Å) operating at 45 kV and 40 mA.

##### 2.2.2.5 Density

The density measurement was performed using the Archimedes’ principle with a Dichtebest Festkoerper FNR 33,360 density testing kit attached to an XR 205SM-DR balance scale. The measurement was performed at room temperature using *n*-heptane as the liquid. The sample density ρ_2_ was calculated as:
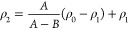
11where ρ_0_ is the density of *n*-heptane (0.6838 g/cm^3^), ρ_1_ is the density of air (0.0012 g/cm^3^), and *A* and *B* are the weights in air and *n*-heptane, respectively.^21^ Three replicates were measured.

## 3 Results and Discussion

### 3.1 Experimental Characterization

**Figure 2:**
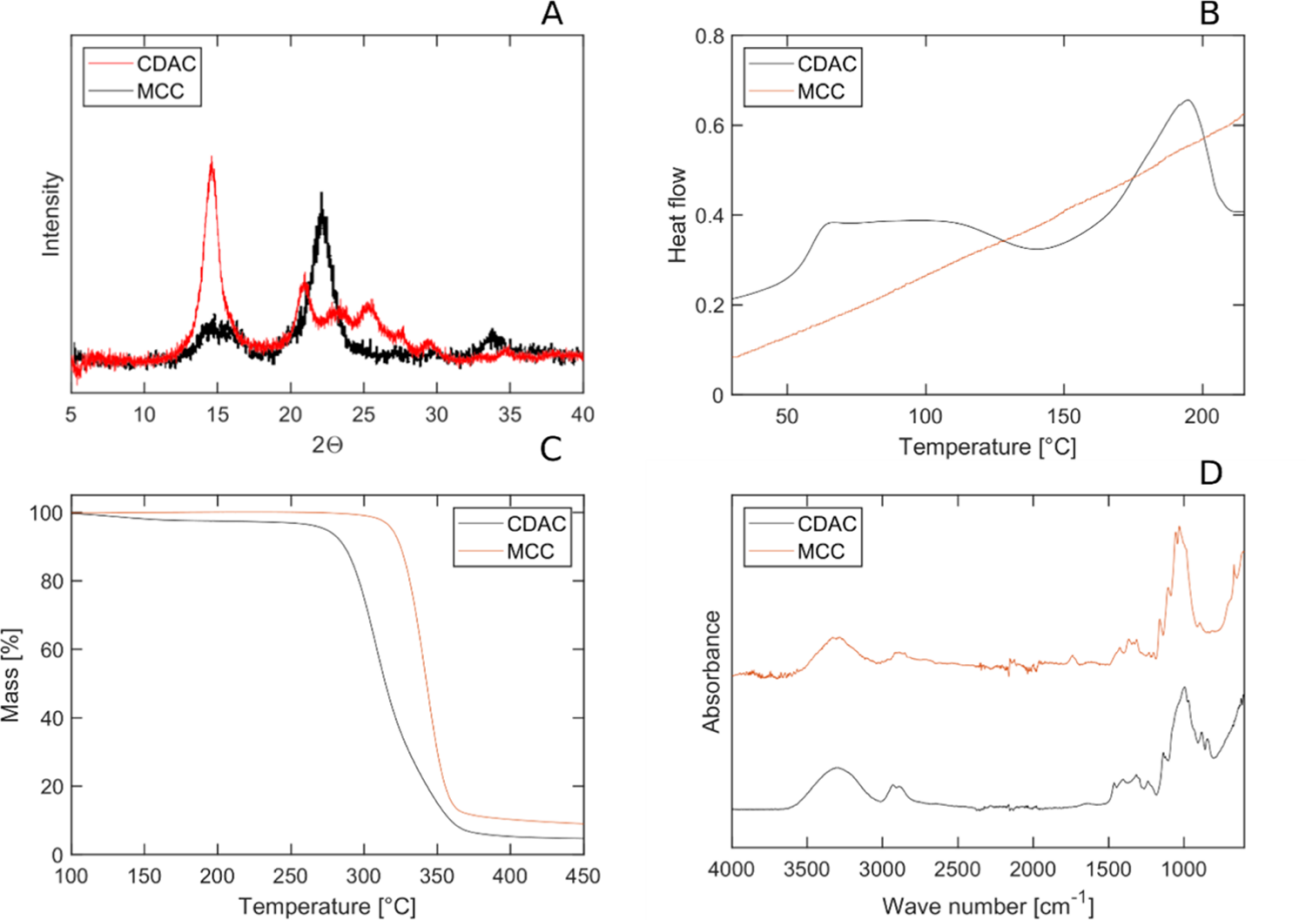
Experimental results for the native cellulose starting material (MCC) and dialcohol cellulose (CDAC) using: (A) XRD, our CDAC measurements, and MCC data adapted from Pachuau et al.^23^ (B) DSC (only second heating shown), (C) Thermogravimetry, and (D) FTIR.

To compare and validate the simulation data, a dialcohol cellulose sample with close to 100% ring opening, based on UV–vis spectroscopy data, was used. The solution-cast film was transparent, which indicates an amorphous material, a low crystallinity, or at least that possible crystallites are significantly smaller than the wavelength of visible light, but XRD indicated a semicrystalline material as seen in Figure 2a, which also explains the relatively high density (1450 kg/m^3^). As mentioned above, DSC revealed that the dialcohol cellulose had a substantially lower glass-transition temperature than cellulose shown in Figures 2b and S3. What was apparent also was that the dialcohol cellulose sample cold-crystallized above the glass-transition temperature (in the approximate region of 120–160 °C), as observed clearly in the second heating seen in Figure S3. The subsequent melting (the peak at approximately 180 °C) involved a larger endothermal change in enthalpy than the exothermal peak preceding it, which indicated that the sample contained crystals after the cooling from the first heating. The low endothermal enthalpy change, ca. 17 J/g, indicated, however, a low overall crystallinity. A small crystallization exotherm was observed in the cooling curve (Figure S3). The first heating curve was less easily interpreted due to a broad endothermal signal due to the evaporation of water. However, the endothermal peak above 200 °C indicated melting of crystals existing in the pristine material and/or formed in the cold-crystallization process; the former supported by the XRD data (Figure 2a). Note the higher melting point than in the second heating. Note also that the second cooling did not show any crystallization. The FTIR spectra shown in Figure 2d of native cellulose and dialcohol cellulose were essentially the same, showing that there were no changes in the functional groups between the materials. However, the thermal degradation occurred at a lower temperature for dialcohol cellulose than for MCC cellulose, which indicated a molar mass reduction accompanying the chemical conversation to dialcohol cellulose seen in Figure 2c.^22^

### 3.2 Simulated Properties

#### 3.2.1 Density, Glass Transition, Thermal Expansion

**Figure 3:**
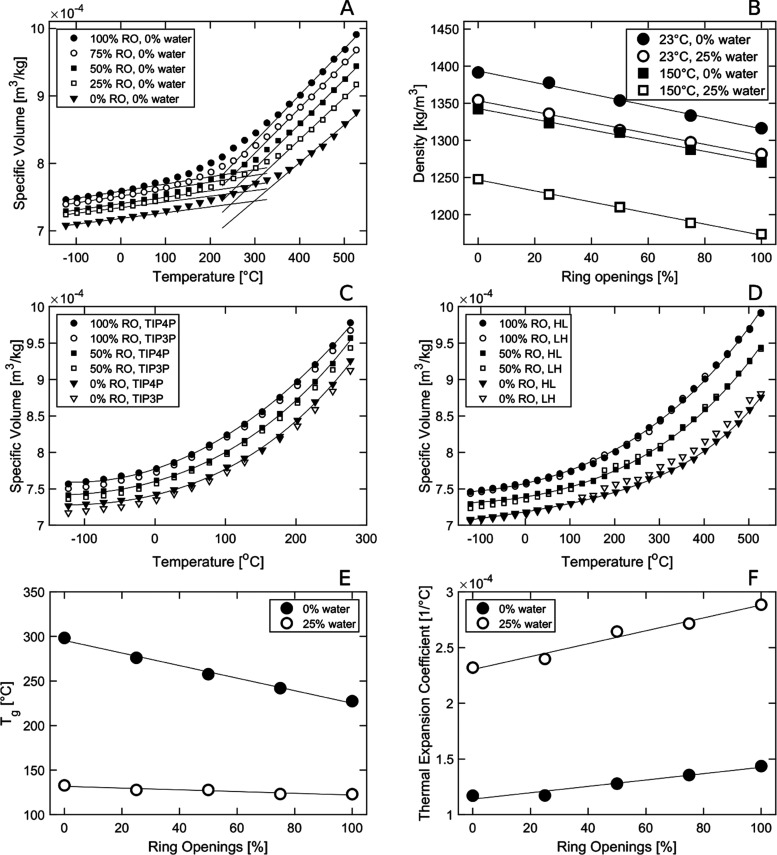
(A) Specific volume as a function of temperature and degree of ring opening (RO) for dry systems. (B) Density versus degree of ring opening and temperature. (C) Specific volume of systems with TIP3P and TIP4P water models in the 25% water systems. (D) Specific volume when heated from low to high temperature (LH) and thereafter cooled from high to low temperature (HL). (E) Glass-transition temperature (*T*_g_) versus degree of ring opening. (F) Thermal expansion coefficient (below *T*_g_) versus degree of ring opening. Line fits show trends in spectroscopy.

The simulated specific volume of the dry systems increased with increasing temperature and degree of ring opening as displayed in Figure 3a. Triplicate simulations showed a small standard deviation of density within each system, which can be seen in Supplementary Information (SI) Figure S1. The maximum standard deviation was 0.24%. The two temperatures of special interest, 23 °C (room temperature) and 150 °C (a temperature that has been used to thermoform dialcohol cellulose fibers),^14^ were analyzed in more detail. This revealed a linear decrease in density with an increasing degree of ring opening shown in Figure 3b, both for the wet (25% water) and dry (0% water) systems. This decrease is due to the formation of the new side groups from the ring opening; each cleaved ring results in two primary hydroxyl groups (and two hydrogen atoms, instead of two secondary hydroxyl groups and a carbon–carbon bond) bound to the main chain, leading to less efficient packing. The density decreased, as expected, also with increasing water content because of a lower density of water, as seen in Figure 3b. The density of the simulated pure cellulose (1390 kg/m^3^ at 23 °C and 0% water) was close to those previously reported for amorphous or paracrystalline cellulose from simulations^24^ and experiments.^25,26^ According to the literature,^27^ crystalline cellulose has a density of 1582–1630 kg/m^3^, while simulated cellulose^24^ has been shown to have a density of 1400–1450 kg/m^3^. However, the density of the simulated (ring-opened) dialcohol cellulose (1320 kg/m^3^) was lower than observed experimentally here (1450 kg/m^3^) at 23 °C and 0% water. This difference in density is most probably a consequence of the presence of a crystalline or “paracrystalline/semiordered” component in the experimental samples, as indicated by the XRD curve with several narrow peaks, with the most prominent occurring around 15° (2Φ), see Figure 2a.

Two common MD models for water are TIP3P and TIP4P, where the TIP3P is optimized for the CHARMM36 force field, which was used in all of the simulations. In Figure 3c, the specific volume–temperature plots, for systems with different degrees of ring opening, are shown with the use of the two water models (25% water). It was observed that the TIP4P water model always yielded higher specific volume than the TIP3P model in the *T*_g_ simulations, but the difference was an average less than 1%. Hence, the computationally cheaper (TIP3P) was used in the study, as recommended for CHARMM36.

To validate that the PVT properties were generated with a “sufficiently” relaxed molecular structure, the PVT curves were generated first from low-to-high (LH) temperature and then from high-to-low (HL) temperature (the latter is also the data in Figure 3a), the latter being standard procedure.^5^ Notably, the most rigid systems (0 and 50% ring opening) showed an upturn in specific volume when heated to the glass-transition region (LH) seen in Figure 3d, most probably due to molecular rearrangement/relaxation when the molecular mobility increased. However, the average difference in specific volume between the LH and HL data for each of the systems was always less than 1%. We observe also the overlapping glassy specific volumes in the LH and HL data for the unmodified system. For moist systems, the differences in specific volumes were even lower, which can be seen in Figure 2S. Hence, the HL-generated PVT data were obtained on sufficiently relaxed molecular systems to provide meaningful specific volumes.

In Figure 3e, we observe that for the simulated systems, a linear decrease in *T*_g_ was observed with increasing degree of ring opening, and the decrease was largest for the dry systems. The simulated *T*_g_ for pure cellulose was ca. 300 °C, which is higher than the experimental value (220–250 °C^28−30^). The difference is expected, considering the very rapid change in temperature in the simulations. Expected was also the significantly lower *T*_g_ in the presence of water (ca. 130 °C, Figure 3e). The simulated *T*_g_ for dry and wet dialcohol cellulose was 220 and 120 °C, respectively. The corresponding experimental values obtained from DSC were 70 and 55 °C, as shown in Figures 3b and S3. Hence, the experimentally observed trend that ring-opened systems have higher molecular mobility was thus predicted by the MD simulations. It should be noted that the specific volume curves for the wet systems (Figure 3c) were more smoothly increasing with increasing temperature than the dry systems (Figure 3a), which were composed of two nearly straight lines and exhibited more distinct glass-transition temperatures. This indicated that the wet systems experienced multiple or broader transitions, as observed experimentally in cellulosic systems.^31^

Figure 3f shows that the simulated thermal expansion coefficient below *T*_g_ increased linearly with increasing degree of ring opening, both for the wet and dry systems. For the wet systems, the slope was steeper and the values were higher. The values are similar or somewhat lower compared to general experimental data of glassy amorphous polymers and water at ambient conditions.^32,33^

#### 3.2.2 X-ray Diffraction Patterns and Radial Distribution Functions

**Figure 4:**
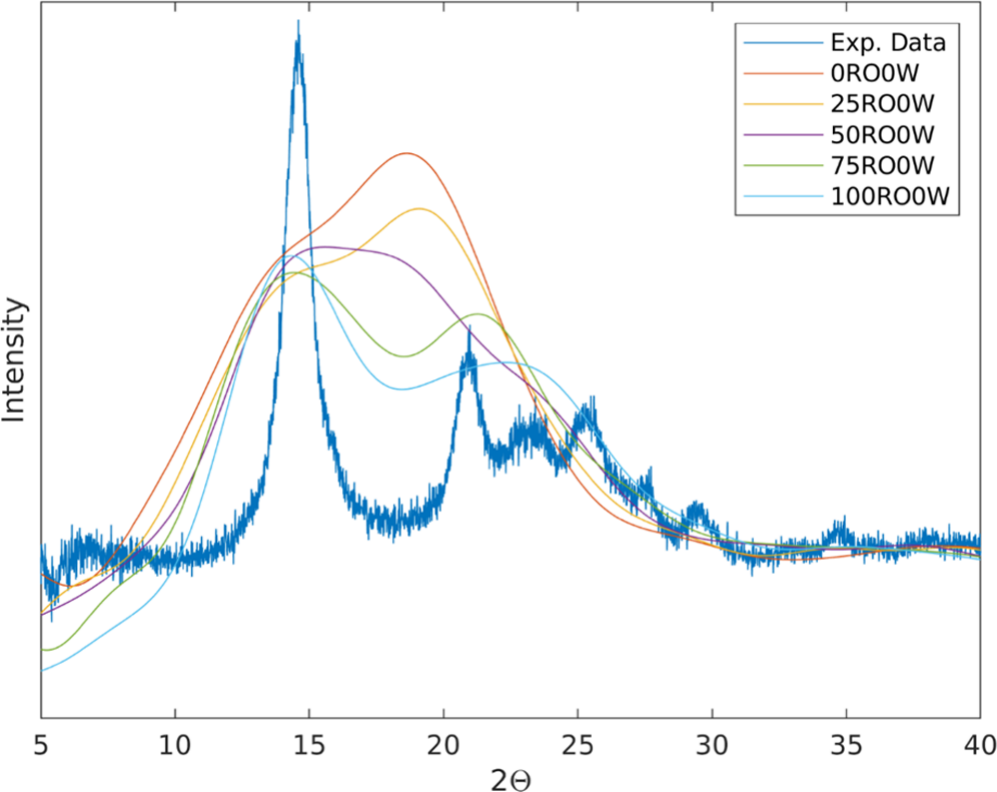
XRD pattern from dry simulations at 23 °C, overlaid on experimental data. The experimental curve is that of 100% ring-opened dialcohol cellulose presented in Figure 2a.

X-ray diffraction (XRD) patterns of simulated materials were obtained using the Debyer software. In Figure 4, XRD spectra for simulated dry (0% RH) materials at 23 °C with 0–100% ring openings are presented, and in Figure S4, additional curves for both wet and dry samples at 23 and 150 °C are shown. The diffraction pattern for the unmodified cellulose exhibits a broad peak with a shoulder, indicating an amorphous structure. For the ring-opened dialcohol cellulose, this peak splits into two more separate peaks around 2θ = 15° and 2θ = 22°, where the former peak increases and the latter decreases in size with increasing degree of ring opening. The higher-angle peak tends to shift toward a higher angle at the same time. These peaks are usually found in crystalline cellulose, where they correspond to the (101) and the (002) planes, respectively.^34^ When comparing simulated and experimental XRD spectra for 100% ring-opened dialcohol cellulose, these peaks nearly coincide, although the experimental curve also has some smaller peaks in the range of 21–30°, as seen in Figure 4. The simulated peaks are, however, significantly broader than those of the experimental material, which are higher and narrower. Thus, the experimental data indicate a semicrystalline material, whereas the simulated materials seem to be amorphous.

Experimentally, for pure cellulose, the peak at 22° normally dominates,^35^ whereas for our 100% dialcohol cellulose, the peak at 15° dominates. Also in the simulations, the height of the 15° peak increases distinctly, whereas the peak around 22° decreases, with increasing degree of ring openings. Since the trend of XRD simulations clearly coincides with the experiments, this is obviously a real consequence of an increased degree of ring openings. This observation could be coupled to experimental findings from the literature, where dialcohol cellulose crystallizes more readily with increasing degree of modification.^36^

The radial distribution function (RDF) for the C4 carbon was used to further investigate the ordering of the atoms,^9,37^ as seen in Figure S5. The height of the first RDF peak, around 5 Å, decreases with increasing degree of ring openings and becomes higher in the presence of water. A comparison with RDF data from Kulasinski^9^ concluded that although there is some ordering in our simulated ring-opened systems, the simulated structures are clearly not crystalline.

#### 3.2.3 Simulated Tensile Properties

**Figure 5:**
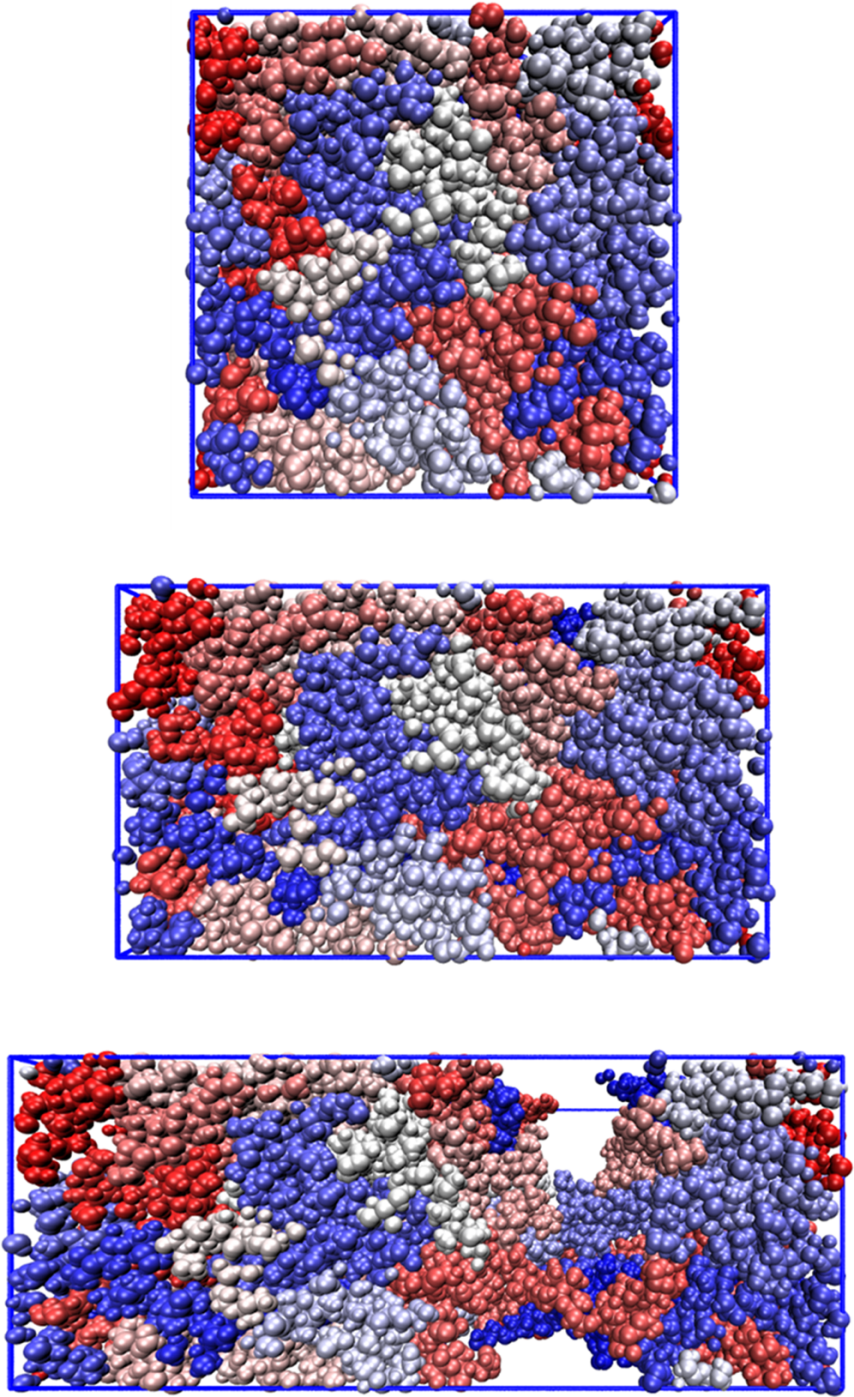
Representative MD boxes of a dry 100% dialcohol cellulose system with 20 polymer chains, each with different color. From top to bottom: 0, 50, and 100% strain. Blue lines represent the extension of the simulation box.

Tensile test simulations were performed to examine the mechanical properties of cellulose with different degrees of ring opening. The tensile test in the molten state can also serve as an indirect assessment of the elongational viscosity in a thermoplastic processing operation. Visualization of a representative dry dialcohol cellulose MD system subjected to 0, 50, and 100% strains reveals that necking (voiding) occurred between 50 and 100% strain, as can be seen in Figure 5. The reduced cross section decreased the force/nominal stress needed for further extension. The presence of 25% water prevented local instability/necking (a material with increased Poisson’s ratio), which can be seen in Figure S6.

**Figure 6:**
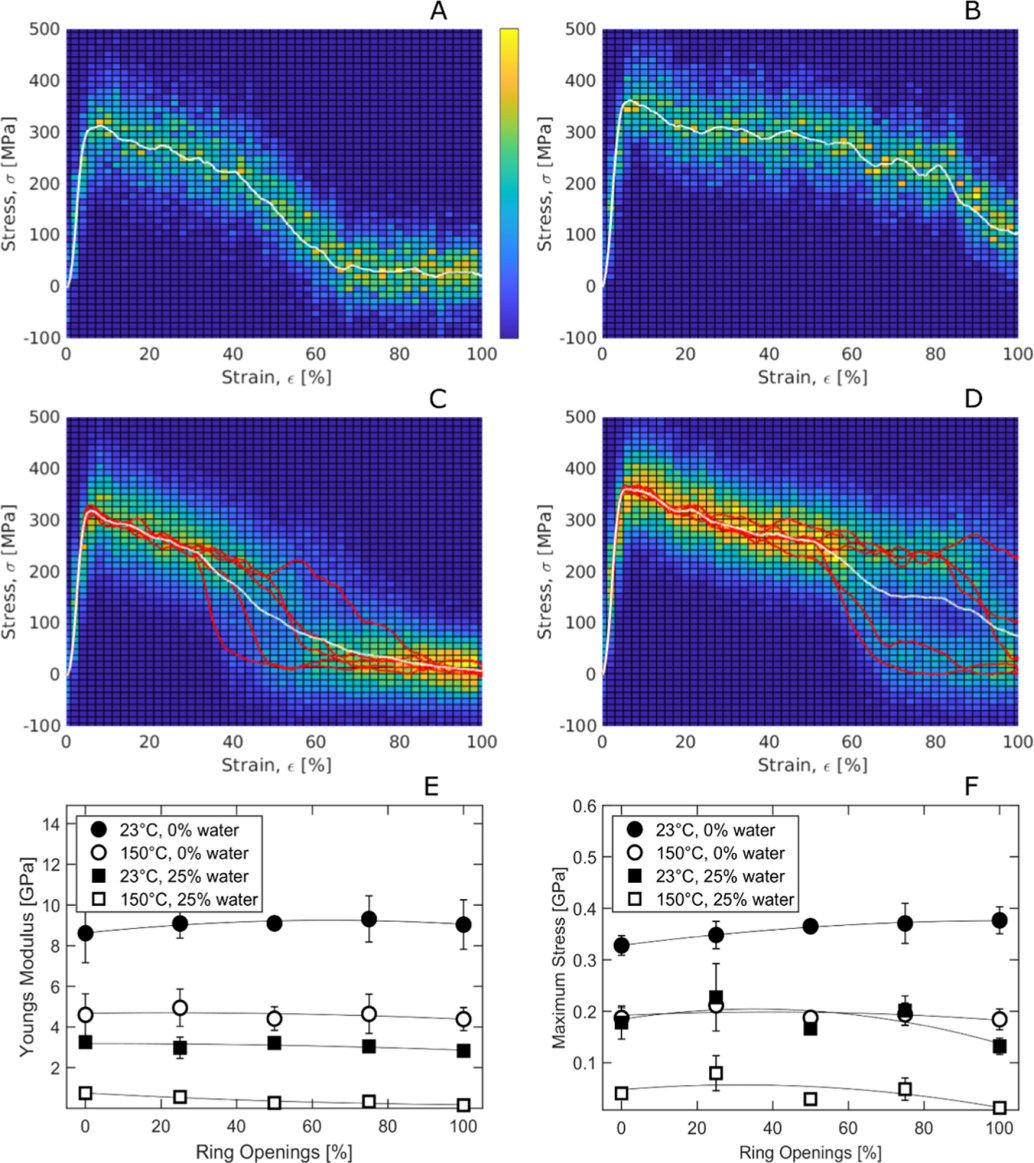
Simulated tensile tests. (A) Single stress–strain curve at 23 °C and 0% water, with 0% ring opening. (B) Same as in (A) but with 100% ring opening. (C) Same as in (A) but with red lines representing stresses at five equidistant slices and white line representing the average. (D) Same as in (C) but with 100% ring opening. (E) Young’s modulus versus degree of ring opening and (F) tensile strength versus degree of ring opening. The light green/blue regions show the scatter in the data.

Stress–strain curves at 23 °C and 25% water content for pure cellulose and pure dialcohol cellulose, i.e., with 100% ring opening, as seen in Figure 6a,b, respectively, indicate a similar initial response of the two materials. However, the stress after the peak drops later for the dialcohol cellulose, indicating a more ductile material after yielding.

GROMACS routines for stress–strain response use stresses on a single face of the computational box. Thus, the response pattern depends on the location of the face and on the void fraction at that position. Since the computational box is periodic, the position of the face can be shifted by translating the atom coordinates in the *x*-direction. When the same boxes as in Figure 6a,b were shifted in five equidistant steps of 20% of the box length, the resulting stress–strain curves differed significantly from each other at large strains, as seen with the red lines in Figure 6c,d. However, the initial response was nearly identical for all curves. Thus, the Young’s modulus and the yield strength of the material can be computed reliably without this shifting, but the stress–strain response at large deformations is preferably computed as the average of several shifts, as seen in Figure 6c,d, white lines.

In Figure 6e,f, we see that both the Young’s modulus and the yield strength decreased with increasing moisture content (plasticization) and temperature, due to the increased molecular mobility.^38^ The simulated modulus for dry cellulose (8.0 ± 0.5 GPa) is close to previously reported values (from modeling).^39^ For the wet systems, the modulus decreased with increasing degree of ring opening (RO), but for the dry systems, it rather increased before starting to decrease, showing a maximum around 50–75% ring opening. The strength showed a similar pattern with respect to the degree of ring opening.

**Figure 7:**
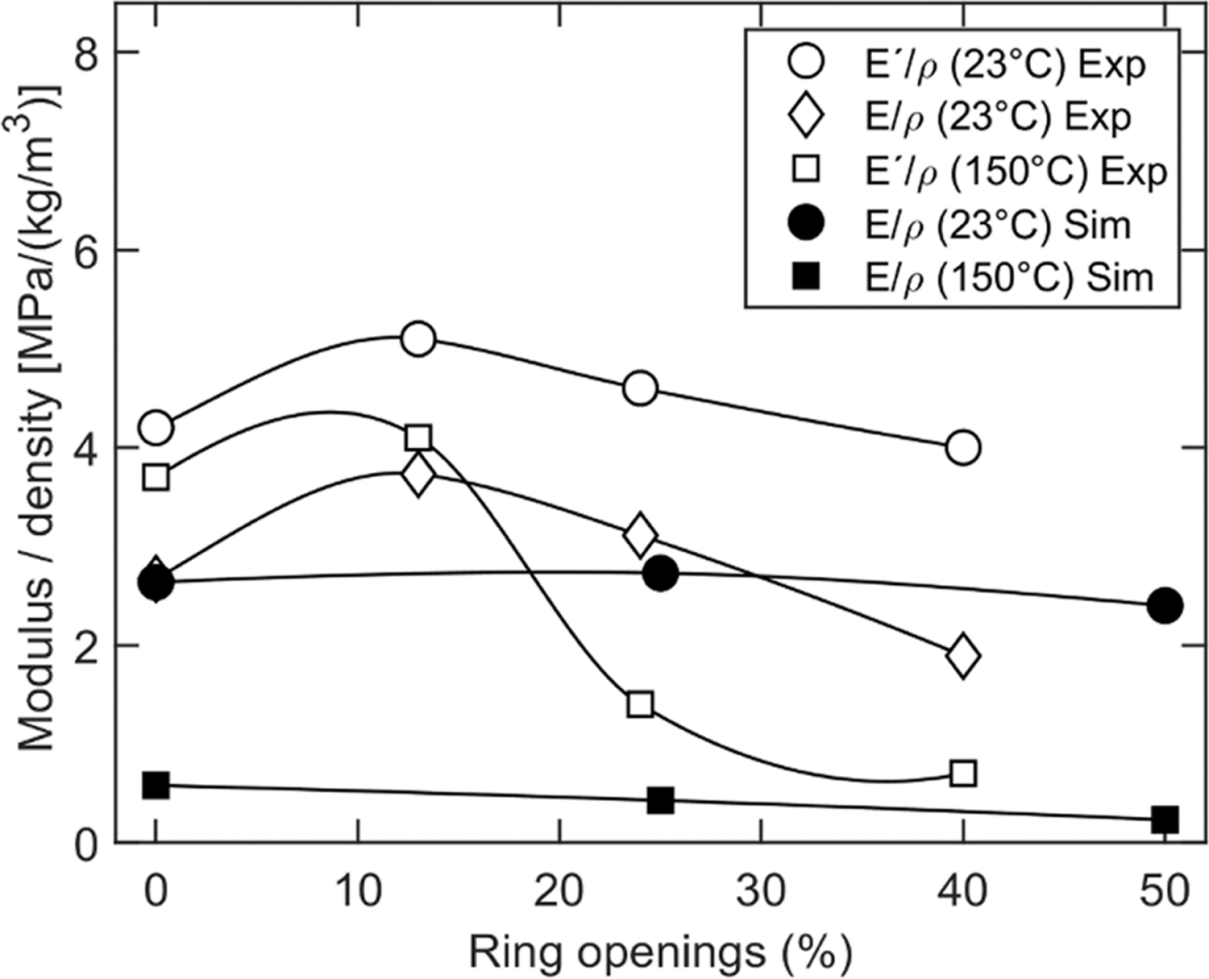
Simulated modulus (25% water) and experimental (measured on sheets made from modified fibers) storage modulus (*E*′) and tensile moduli (*E*), normalized to the density of the material (ρ)^14^ as a function of degree of ring opening.

In Figure 7, the density-normalized simulated modulus was compared with experimental tensile (Young’s) modulus and storage/dynamic modulus from dynamic mechanical analysis (DMA).^14^ It should be noted that the systems are quite different in terms of morphology. The simulated system is 100% amorphous, whereas the experimental systems contain fibers/nanofibrils that have been partly converted to dialcohol cellulose, i.e., still consisting of a fraction of crystalline cellulose. The initial moisture content in the real material is also intermediate to that of the simulated systems. Nevertheless, the trends show some common important features. Within the range of experimental dialcohol cellulose content (0–40%) and simulated range (0–100%), both the simulated and experimental data indicate a clear decrease in modulus/stiffness with increasing degree of ring opening at 150 °C, whereas the decrease is less, or even absent, at room temperature (Figures 6e and 7). Hence, the increase in molecular mobility after ring opening is more noticeable above (experimental data, Figure 2b) and in the vicinity (simulated data, Figure 3e) of the glass-transition temperature region. There are several reasons why the simulated data in Figure 7 were on the same level or even lower than those from experiments. The expected increase in modulus in simulations due to the very high strain rate is often compensated for by that the chains are shorter in simulations than in the real material (chain-end effects^10,40^). In addition, the wet simulated systems contained a larger amount of water. Finally, it should also be noted that the simulated modulus and strength were determined by considering the actual box cross section, which means that up to the yield point, they corresponded to true stress, whereas the experimental data were based on the initial sample cross section, i.e., engineering stress. However, this effect is small at a strain up to the yield stress, as seen in Figure 6.

#### 3.2.4 Hydrogen-Bond Density and Potential Energy

**Figure 8:**
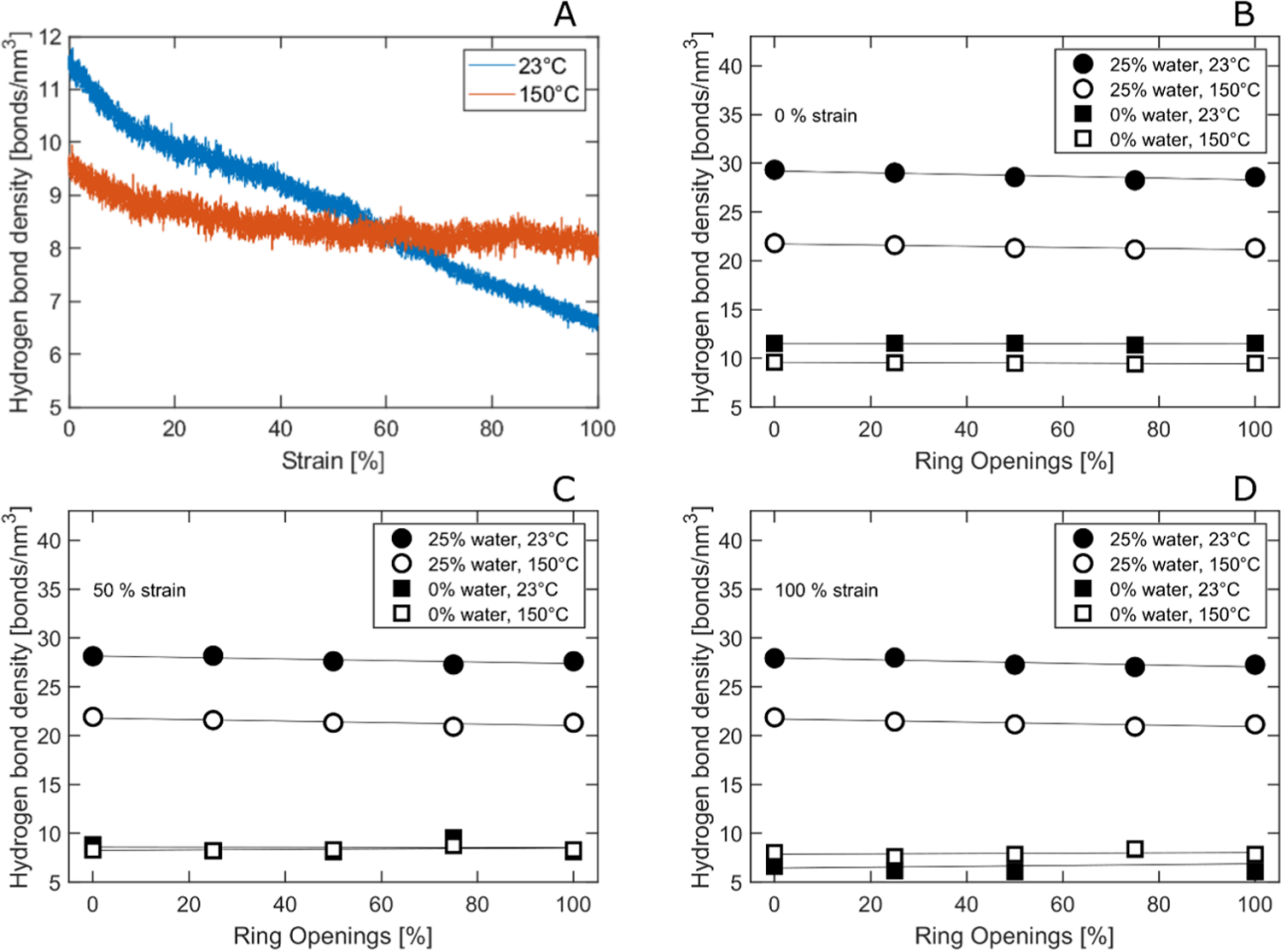
(A) Hydrogen-bond density versus strain and temperature (pure, dry cellulose). (B–D) Hydrogen-bond density versus degree of ring opening at 0% strain at different temperatures and water contents. (C, D) Same as (B) at 50 and 100% strain, respectively.

In Figure 8a, we see that for pure dry cellulose, the total hydrogen-bond density decreased with increasing strain, both at 23 and 150 °C. The decrease was, however, less pronounced at higher temperatures, indicating that the molten material, with its higher molecular mobility, favored the formation of new or reorienting hydrogen bonds during tensile deformation. The voiding/necking would also decrease the hydrogen-bond density. At all strains (0, 50, and 100%), the hydrogen-bond density increased in the presence of water, and in these systems, the hydrogen-bond density was always higher at lower temperatures (23 °C), as seen in Figure 8b–d and Table 1. As the fraction of oxygen atoms is the same in both cellulose and dialcohol cellulose, the hydrogen-bond density was not changing significantly with the degree of ring opening. Only a small linear decrease was observed with increasing degree of ring opening in the water-containing systems and in the undeformed dry systems slightly (linearly) with increasing degree of ring openings.

**Table 1:** Hydrogen-Bond Lifetimes (ns) and Hydrogen-Bond Density (Hydrogen Bonds/nm^3^)

lifetime				density			
23 °C^a^	PP^b^	PW^b^	WW^b^	PP^b^	PW^b^	WW^b^	total
0RO0W	41,000			11.4 ± 0.1			11.4 ± 0.1
50RO0W	51,000			11.5 ± 0.1			11.5 ± 0.1
100RO0W	32,000			11.4 ± 0.1			11.4 ± 0.1
0RO025W	16,000	>350	110	4.9 ± 0.1	12.8 ± 0.1	11.6 ± 0.1	29.4 ± 0.3
50RO25W	18,000	>410	150	5.0 ± 0.1	12.4 ± 0.1	11.2 ± 0.1	28.6 ± 0.3
100RO25W	11,000	>390	160	4.6 ± 0.1	13.1 ± 0.2	10.4 ± 0.1	28.0 ± 0.3

150 °C	P	PW	W	P	PW	W	Total
0RO0W	19,000			9.6 ± 0.1			9.6 ± 0.1
50RO0W	9000			9.5 ± 0.1			9.5 ± 0.1
100RO0W	7000			9.4 ± 0.1			9.4 ± 0.1
0RO025W	3900	12	10	4.1 ± 0.1	8.9 ± 0.2	9.0 ± 0.1	22.0 ± 0.4
50RO25W	3200	11	10	4.0 ± 0.1	8.8 ± 0.2	8.6 ± 0.1	21.4 ± 0.4
100RO25W	700	10	10	3.5 ± 0.1	9.1 ± 0.2	7.9 ± 0.1	20.5 ± 0.4

a 0RO0W means 0% ring opening and 0% water content.

b PP polymer–polymer, PW: polymer–water, WW: water–water hydrogen bond.

The polymer–polymer hydrogen-bond lifetimes (τ_HB_) were several orders of magnitude longer than the polymer–water and water–water hydrogen bonds (Table 1). This is due to the tightly packed nature and stiffness/rigidity of the polymer chains, making hydroxyl groups less mobile and hydrogen bonds more stable. For the polymer–polymer interactions, especially in the dry systems at 23 °C, the scatter in τ_HB_ between different amorphous configurations was pronounced. The τ_HB_ involving water was much shorter than for the dry systems (Table 1). At high temperatures, the water moves quickly and rarely creates long-lasting hydrogen bonds. For wet systems at 150 °C, τ_HB_ decreased with increasing degree of ring opening because of the higher mobility of the polymer chains.

**Figure 9:**
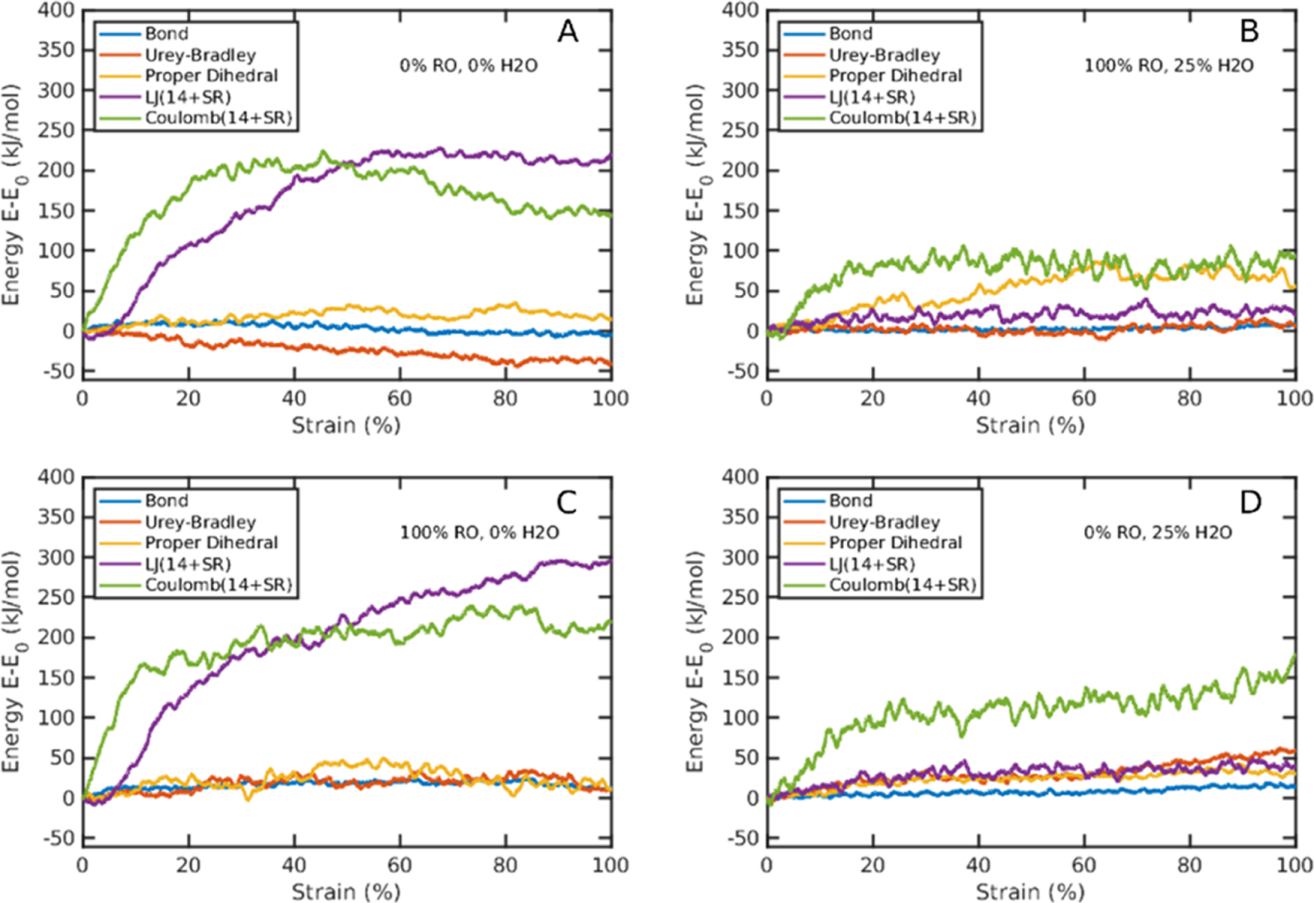
(A–D) Potential energy versus strain at different degrees of ring opening (RO) and water content. The contributions are bond vibrations, molecular bending (Urey–Bradley), improper dihedral torsions, Lennard-Jones (LJ) contributions, and Coulomb interactions.

The total potential energy in the polymer system was strongly affected by the applied strain seen in Figure 9. For dry systems, both with 0 and 100% ring openings (Figure 9a,c), a pronounced increase in intermolecular forces (Lennard-Jones (LJ) and Coulomb contributions) was observed, especially up to 10–50% strain. This corresponds well with the observations in the corresponding stress–strain curve simulations. A pronounced increase in Coulomb energy was observed also for the wet systems, although to lower levels. Hence, the presence of water (and its plasticizing effect) preserves, to a large extent, the atomistic packing during the mechanical deformation, as also demonstrated in Figure S6. The intramolecular energies (bond, bend (Urey–Bradley), and torsional/dihedral) were affected, in general, less than the Coulombic energy and in the dry system also the LJ energy during the deformation. However, noteworthy is that the bend energy decreased in the dry cellulose (0% ring opening) with increasing strain. This is probably due to a relaxation of the molecules associated with an increase in volume (voiding) during the deformation. This behavior in bend energy has also been observed earlier for a starch/glycerol system.^10^

#### 3.3.3 Diffusivity and Free Volume

**Figure 10:**
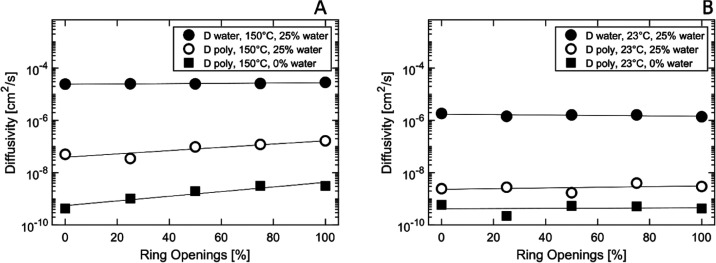
(A, B) Simulated diffusivity of water and cellulose/modified cellulose as a function of degree of ring opening at different temperatures. The diffusivity is computed from the slope of the mean square displacement curves using Einstein’s method.

In Figure 10, we see that the polymer (self) diffusivity at 150 °C was ca. 1 × 10^–7^ cm^2^/s for the wet cellulose systems and 2 orders of magnitude lower (ca. 1 × 10^–9^ cm^2^/s) for the dry systems (Figure 9). The increased mobility of the wet systems is due to the plasticizing effect of water in hydrophilic polymers with a large content of hydroxyl groups, such as cellulose and dialcohol cellulose.^41,42^ A linear increase in polymer diffusivity was observed with increasing degree of ring opening, with a total increase of approximately 1 order of magnitude. The water diffusivity at 150 °C was nearly independent of the degree of ring opening, ca. 3 × 10^–5^ cm^2^/s. The TIP3P water model has a boiling temperature of 320 °C,^5^ but since the mobility of the water molecules would increase even more in the gas phase, all qualitative conclusions remain valid.

At 23 °C, the diffusivity of the polymer was, as expected, lower than that at 150 °C, but it was still linearly dependent on the degree of ring opening, which can be seen in Figure 10. However, only a slightly positive slope was observed for the wet systems, and for the dry systems, the slope was even negative. At 23 °C the water diffusivity was approximately 1 order of magnitude lower (2 × 10^–6^ cm^2^/s) than at 150 °C. The diffusion coefficients of these solid materials are higher than what would be seen experimentally, but the trends of increasing diffusivity with ring openings are still notable.

**Figure 11:**
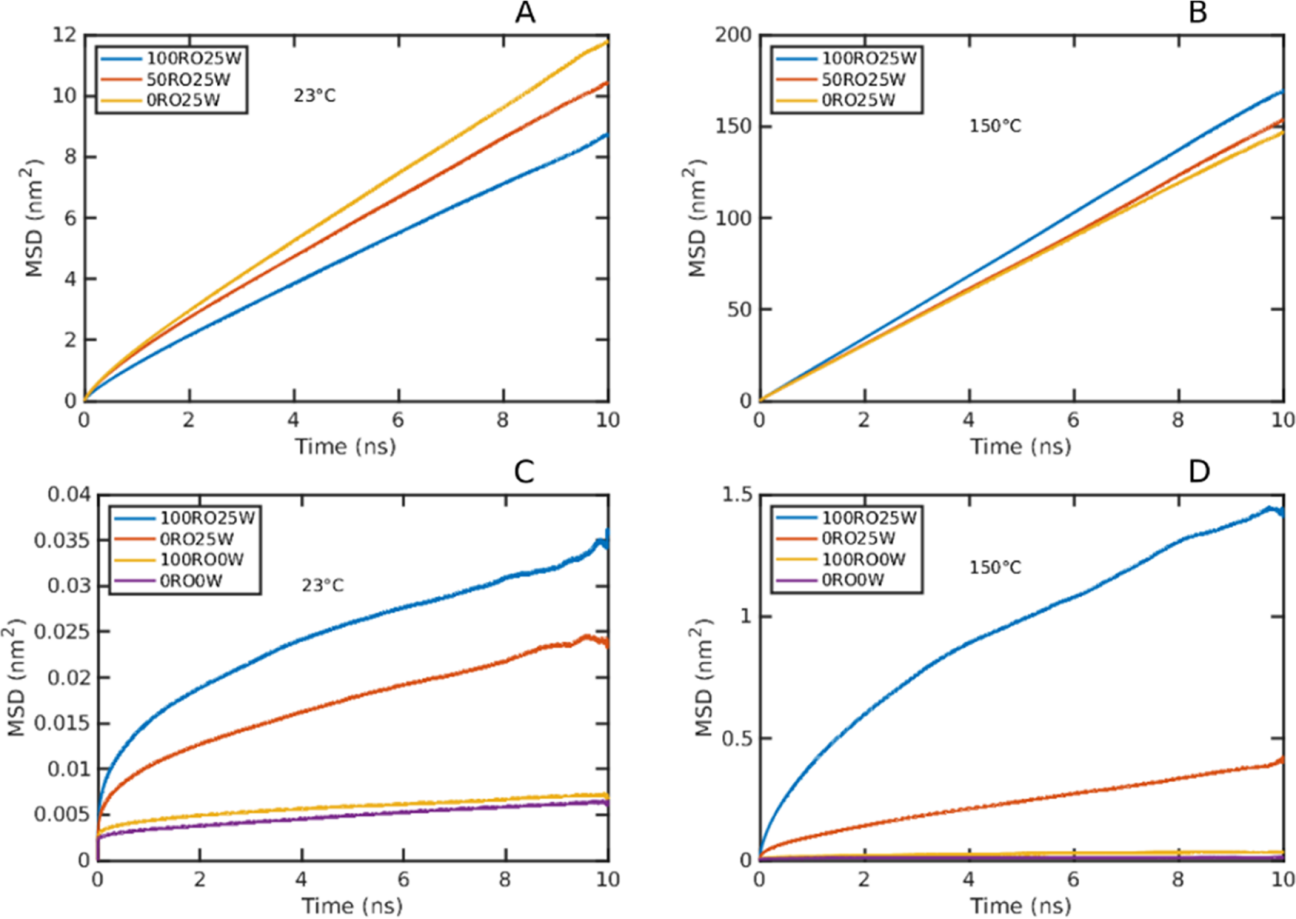
Mean square displacement versus time for (A) water, 23 °C, (B) water 150 °C, (C) polymer, 23 °C, and (D) polymer, 150 °C.

The diffusivity was obtained from the linear or near-linear part of the mean square displacement (MSD) curves using eq 7. The MSD curves of water were linear at 150 °C or nearly linear at 23 °C, as seen in Figure 11a,b, whereas those of the polymer exhibited an initially nonlinear trend, typical for cage-like diffusion, as seen in Figure 11c,d.^5^ Therefore, the diffusivity of the polymer systems was assessed from the most linear parts of the curve, e.g., between 3 and 7 ns, where the MSD refers better to the random walk process.

**Figure 12:**
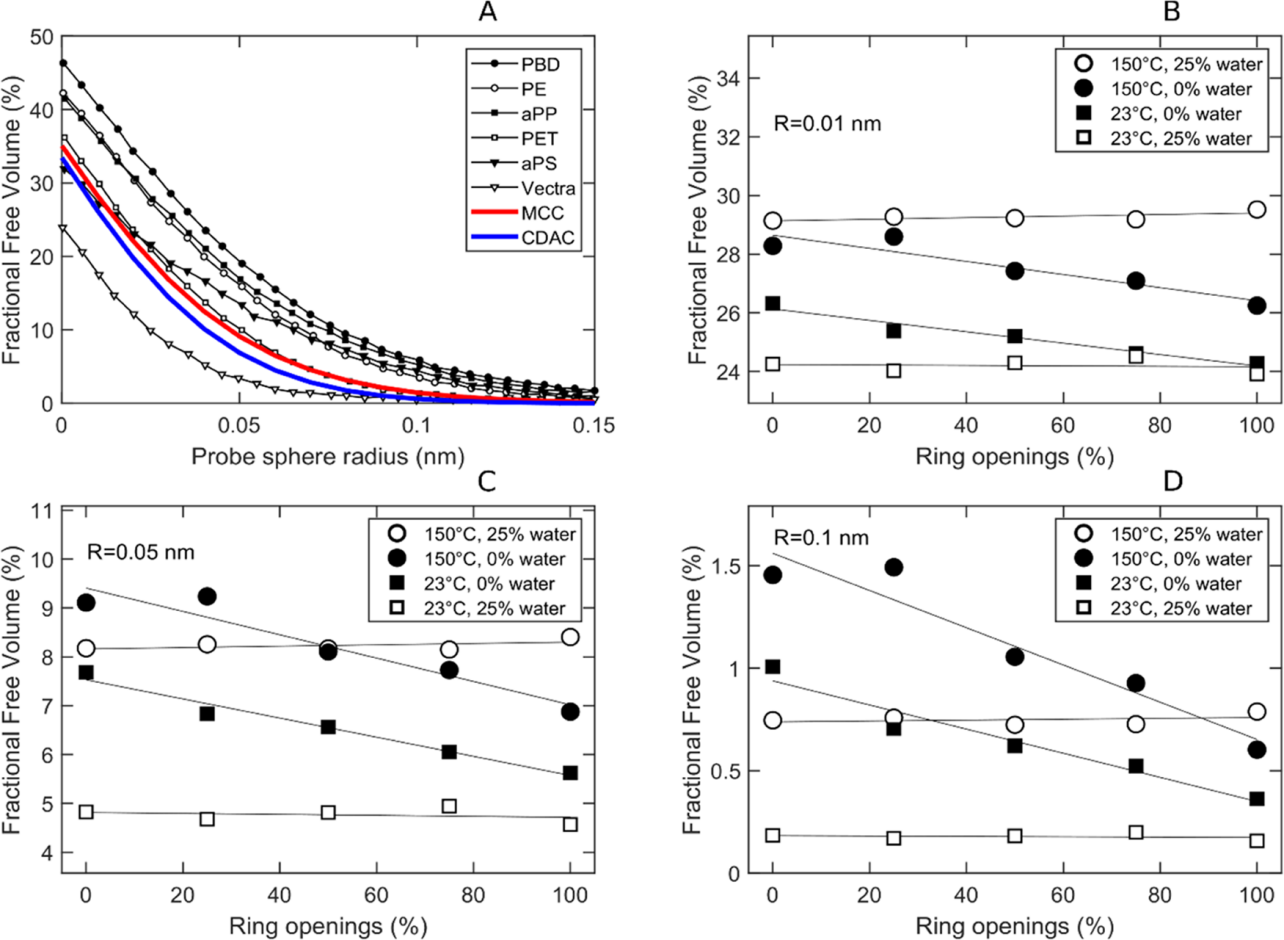
Fractional free volume (FFV). (A) FFV of cellulose and other polymers from the literature (data adapted from Boyd et al.^43^). FFV versus degree of ring opening at different temperatures and humidity, using a probe radius of (B) 0.01 nm, (C) 0.05 nm, and (D) 0.1 nm.

An important factor affecting diffusivity/mobility is the size and size distribution of the free volume in the system. In Figure 12a, we see that the fractional free volume of the amorphous cellulose system, plotted as a function of probe sphere radius, was somewhat lower than for typical commodity polymers (amorphous simulated systems), including polyethylene (PE), polypropylene (PP), poly(ethylene terephthalate) (PET), and polystyrene (PS). Only the liquid crystalline polymer (Vectra), which is a very good gas and liquid barrier material, had a lower fractional free volume.^43^ This indicates that the material can potentially be a good barrier material with low diffusivity for many penetrant molecules.^14,44^ The fractional free volume distribution of cellulose, with or without ring opening and water and independent of temperature, was also similar to that of the other polymers, with only polystyrene showing a different distribution (Figures 11a and S6). In dry conditions, the free volume at a 0.01 nm probe radius decreased slightly with increasing degree of ring opening, whereas it remained nearly constant in wet conditions seen in Figure 12b. Due to thermal expansion, the free volume increased at higher temperatures. For all probe radii, the wet 23 °C system had the lowest free volume because the water molecules were well distributed over the box volume. However, in Figure 12c,d, we see that at 150 °C, the wet system had sometimes a higher free volume than the dry system, depending on the actual probe radius and degree of ring opening. This is probably due to the unoccupied space inside the cellulose rings, which cannot host probes and molecules above a certain radius.

## 4 Conclusions

Dry and wet amorphous systems containing amorphous cellulose and dialcohol cellulose were simulated with molecular dynamics in order to examine how and why the degree of ring openings influences the thermoplastic and mechanical properties of cellulose and dialcohol cellulose. As complement and validation for the simulations, experimental measurements were performed on such materials. The goal of the study was to understand how improved thermoplastic bioplastics can be derived from cellulose-containing natural sources like wood.

The mobility, fractional free volume, and polymer diffusivity of the cellulose systems were all affected by the presence of water, the actual temperature, and degree of ring opening. These effects correlated with changes in molecular interactions/potential energy and hydrogen-bond density and lifetime, which in turn affected the mechanical properties of the materials. As expected, the mobility increased with increasing temperature and water content, leading to lower elastic modulus and strength but higher polymer diffusivity and ductility (and consequently higher thermoplasticity). The simulated density and *T*_g_ (as well as the experimental *T*_g_) decreased with increasing degree of ring opening, whereas diffusion and tensile simulations revealed more complex patterns. However, the simulations showed what was observed in the experiments, that the impact of ring opening toward a more “processable” material (lower stiffness and yield strength) was greater at a high temperature (150 °C). Hence, the simulations showed that the conversion from cellulose to dialcohol cellulose provided increased molecular mobility at conditions where thermoplastic processing normally is performed (above *T*_g_) but has less effect on the material/mechanical properties at ambient conditions. The findings in this study reveal trends and molecular mechanisms that are valuable to assess for the development of thermoplastic polymers from, e.g., wood-based natural resources.

## Supporting Information Available

Additional data for density, equilibration scheme, force-field parameters, and additional plots and graphics (PDF)
