# Supplementary material for: Molecular Dynamics Simulations of Cellulose and Dialcohol Cellulose under Dry and Moist Conditions
Source: Biomacromolecules. 2023 May 11;24(6):2706–20. doi: 10.1021/acs.biomac.3c00156 (PMC10265663; doi:10.1021/acs.biomac.3c00156)
Supplement: Supplementary file 1 — bm3c00156_si_001.pdf [file bm3c00156_si_001.pdf]

Supplementary Information

Molecular Dynamics Simulations of Cellulose and Dialcohol Cellulose in Dry and Moist Conditions

Patric Elf[1,3], Hüsamettin Deniz Özeren[6], Per Larsson[1,3], Anette Larsson[2,4], Lars Wågberg[1,3], Robin Nilsson[2,4], Poppy Chaiyupatham[2], Mikael S. Hedenqvist[1,3*], Fritjof Nilsson[1,5*]

[1]School of Engineering Sciences in Chemistry, Biotechnology and Health, Fibre and Polymer Technology, KTH Royal Institute of Technology, SE-100 44 Stockholm, Sweden

[2]Department of Chemistry and Chemical Engineering, Chalmers University of Technology, SE-412 96 Gothenburg, Sweden

[3]FibRe Centre for Lignocellulose-based Thermoplastics, KTH Royal Institute of Technology, Stockholm, Sweden

[4]FibRe Centre for Lignocellulose-based Thermoplastics, Chalmers University of Technology, SE-412 96 Gothenburg, Sweden

[5]FSCN research centre, Mid Sweden University, 85170 Sundsvall, Sweden

[6] Division of Glycoscience, Department of Chemistry, AlbaNova University Centre, KTH Royal Institute of Technology, SE-106 91 Stockholm, Sweden

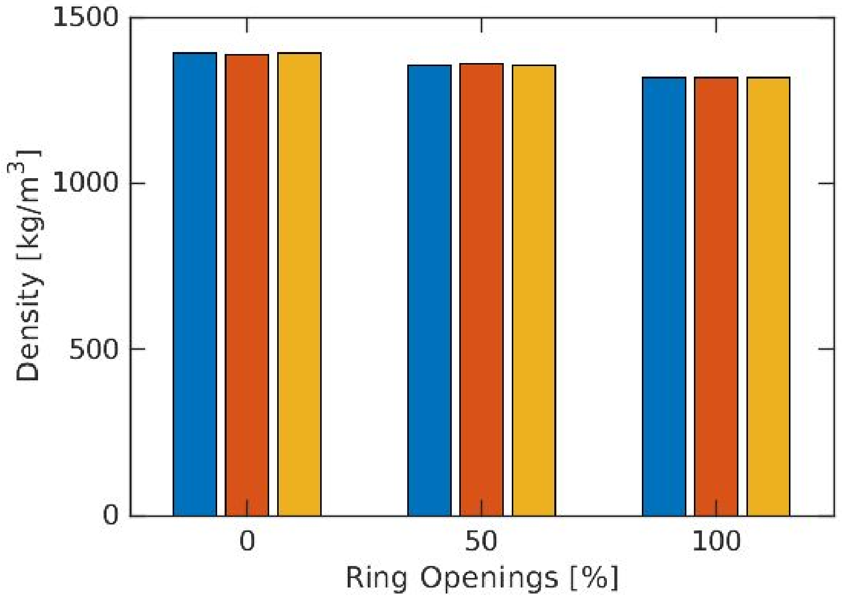

Figure S1. Density of dry material with different degrees of ring-opening at 23 °C as triplicates.

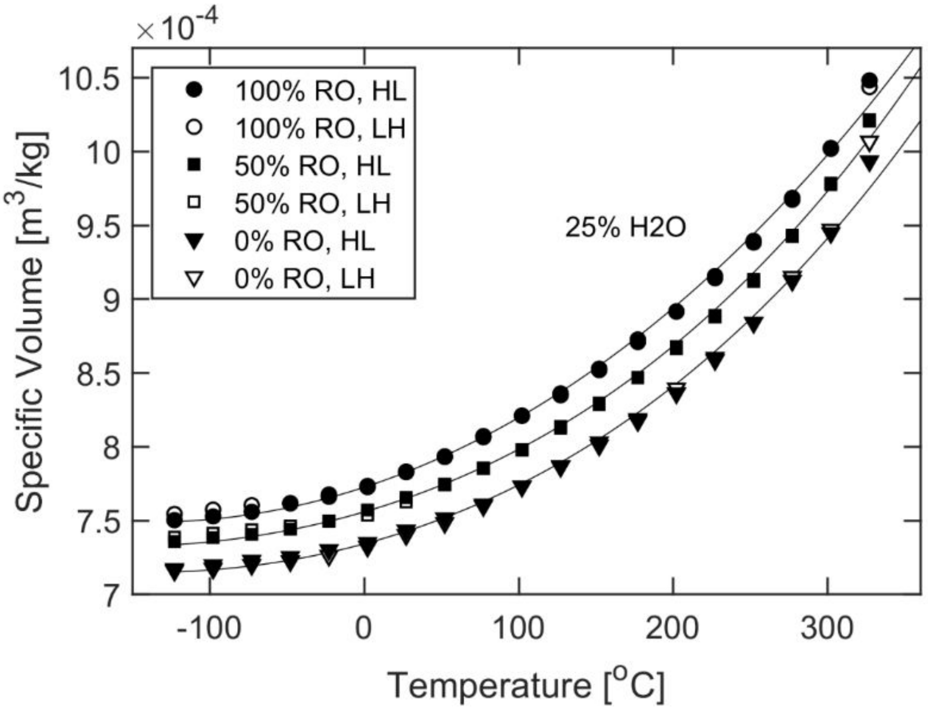

Figure S2. Specific volume versus temperature and degree of ring opening for 25 % water systems starting from low-to-high (LH) and then high-to-low (HL) temperatures, respectively.

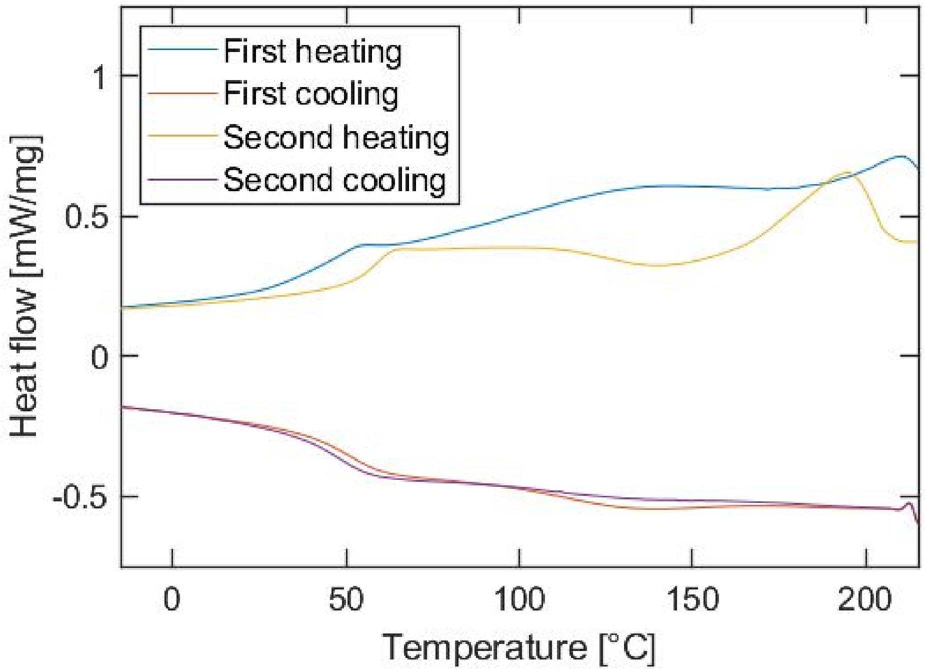

Figure S3. Heating and cooling thermograms of the dialcohol cellulose film. Endothermal signal is up.

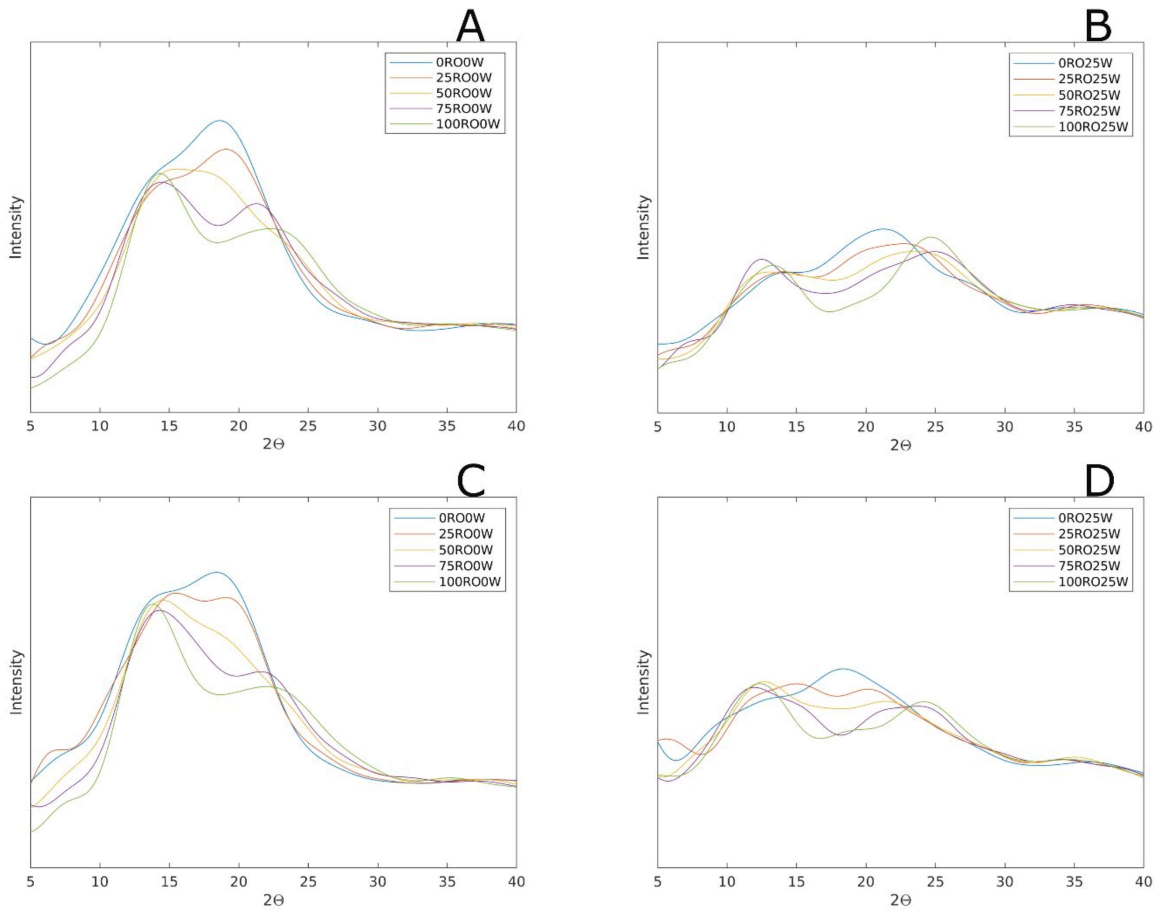

Figure S4. Simulated XRD-spectra for different degrees of ring-openings between 0 and 100 %. **(a)** 23 °C, dry **(b)** 23 °C, wet **(c)** 150 °C, dry **(d)** 150 °C, wet.

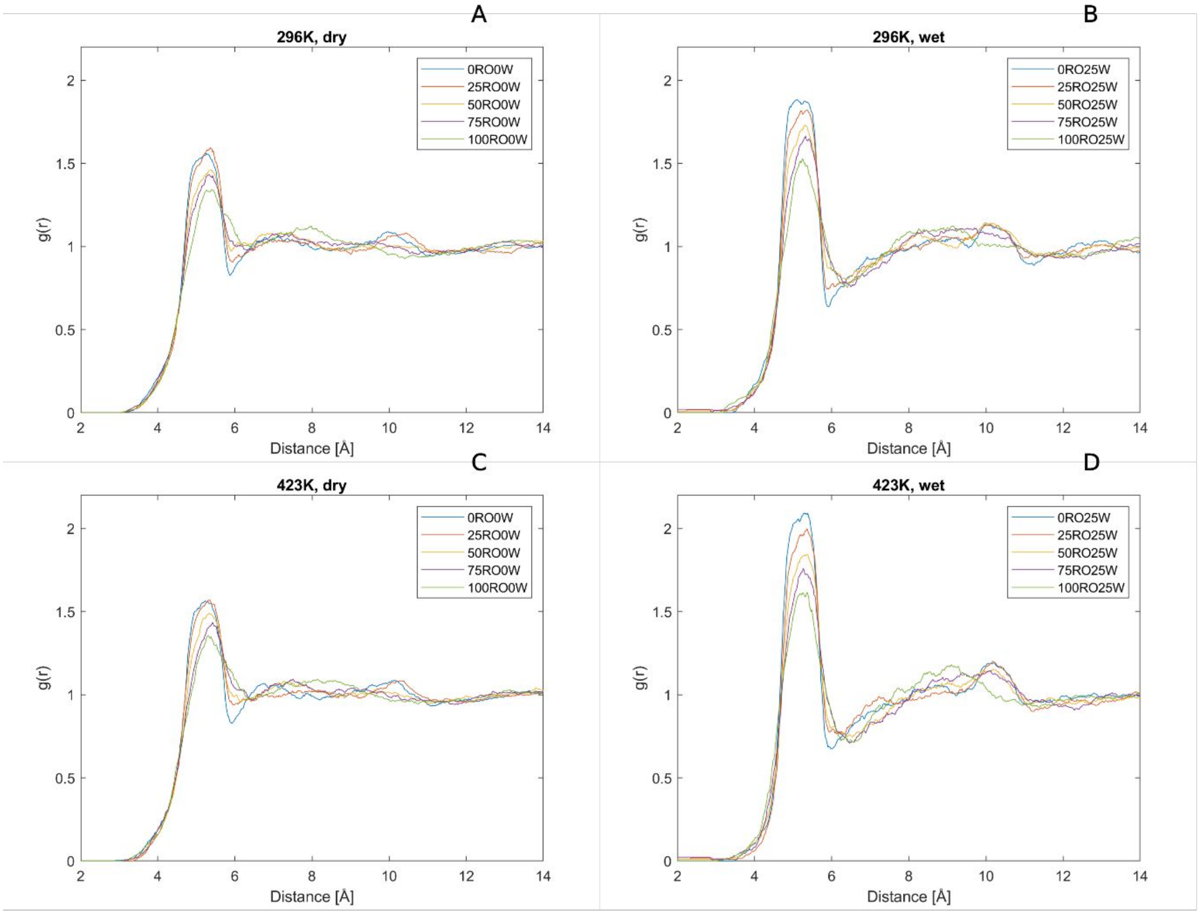

Figure S5. Radial distribution functions of the C4 atoms, smoothed curves, **(a)** 23°C, dry **(b)** 23°C, wet **(c)** 150°C, dry **(d)** 150°C, wet.

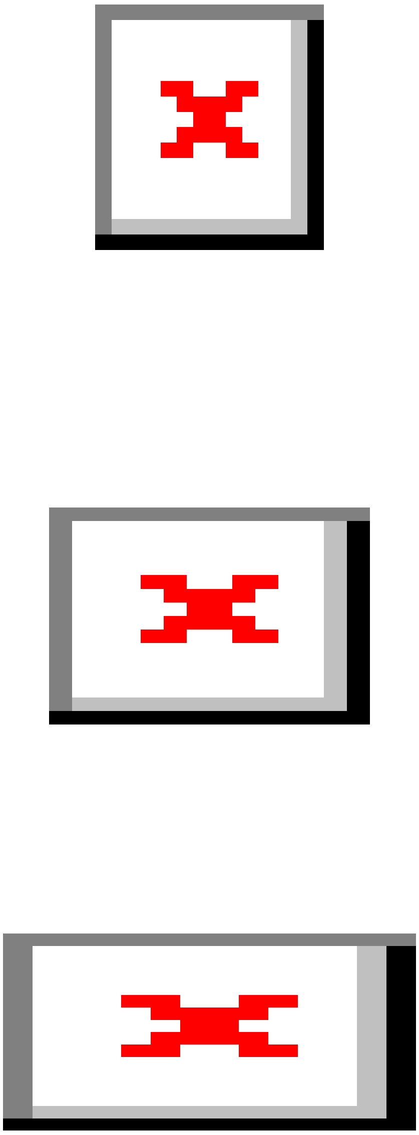

Figure S6. Representative MD boxes of a wet 100% dialcohol cellulose system with 16 polymer chains, each with different color. From top to bottom: 0, 50 and 100 % strain.

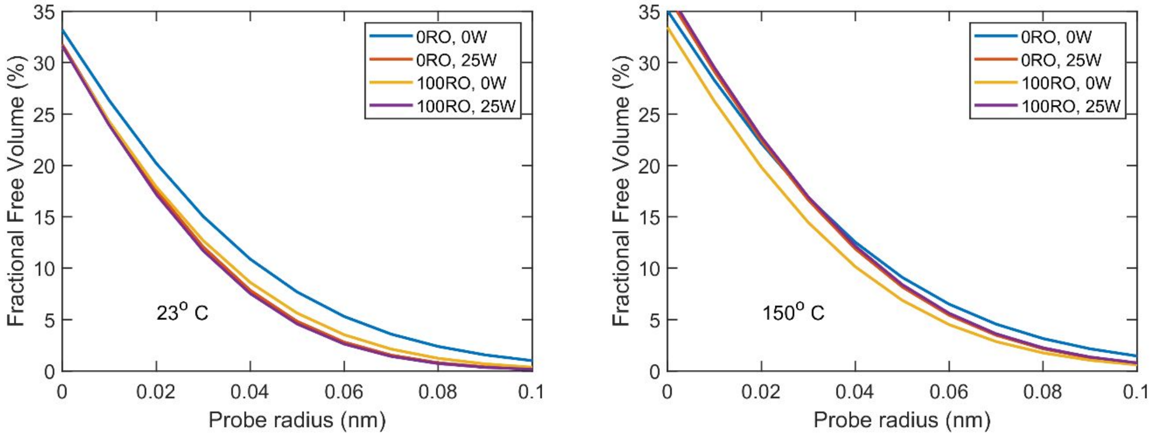

Figure S6. Fractional free volume versus probe radius, humidity and temperature.

A modified version of the CHARMM36-feb2021.ff forcefield was used. An .rtp file containing the residues was added, as well as definitions to the “ffbonded.itp” file. The charge distribution for the cellulose residue types was taken from the CHARMM36 Glycan Reader & Modeler charges, with slight modifications for the dialcohol cellulose residue to have a net zero charge for each polymer chain. The definitions added in “ffbonded.itp” was taken as similar angles/dihedrals already present in the CHARMM36 force field, just with the atomtypes replaced.

Table S1. Charge distribution, derived from CHARMM36 with minor modifications. BLC, GLC and TLC are the beginning, middle and terminal unit of the cellulose polymer chain, and BL1, GL1 TL1 are the corresponding ones for the units in the dialcohol cellulose polymer chains.

	BLC			GLC			TLC		
No.	Atom	Atomtype	Charge	Atom	Atomtype	Charge	Atom	Atomtype	Charge
0	C1	CC3162	0.34	C1	CC3162	0.29	C1	CC3162	0.29
1	H1	HCA1	0.09	H1	HCA1	0.09	H1	HCA1	0.09
2	O1	OC311	-0.65	C5	CC3163	0.11	C5	CC3163	0.11
3	HO1	HCP1	0.42	H5	HCA1	0.09	H5	HCA1	0.09
4	C5	CC3163	0.11	O5	OC3C61	-0.4	O5	OC3C61	-0.4
5	H5	HCA1	0.09	C2	CC3161	0.14	C2	CC3161	0.14
6	O5	OC3C61	-0.4	H2	HCA1	0.09	H2	HCA1	0.09
7	C2	CC3161	0.14	O2	OC311	-0.65	O2	OC311	-0.65
8	H2	HCA1	0.09	HO2	HCP1	0.42	HO2	HCP1	0.42
9	O2	OC311	-0.65	C3	CC3161	0.14	C3	CC3161	0.14
10	HO2	HCP1	0.42	H3	HCA1	0.09	H3	HCA1	0.09
11	C3	CC3161	0.14	O3	OC311	-0.65	O3	OC311	-0.65
12	H3	HCA1	0.09	HO3	HCP1	0.42	HO3	HCP1	0.42
13	O3	OC311	-0.65	C4	CC3161	0.09	C4	CC3161	0.14
14	HO3	HCP1	0.42	H4	HCA1	0.09	H4	HCA1	0.09
15	C4	CC3161	0.09	O4	OC301	-0.36	O4	OC311	-0.65
16	H4	HCA1	0.09	C6	CC321	0.05	HO4	HCP1	0.42
17	O4	OC301	-0.36	H61	HCA2	0.09	C6	CC321	0.05
18	C6	CC321	0.05	H62	HCA2	0.09	H61	HCA2	0.09
19	H61	HCA2	0.09	O6	OC311	-0.65	H62	HCA2	0.09
20	H62	HCA2	0.09	HO6	HCP1	0.42	O6	OC311	-0.65
21	O6	OC311	-0.65				HO6	HCP1	0.42
22	HO6	HCP1	0.42						
	Bonds			Bonds			Bonds		
	C1	O1		C1	H1		C1	H1	
	C1	H1		C1	O5		C1	O5	
	O1	HO1		C1	C2		C1	C2	
	C1	O5		C2	H2		C2	H2	
	C1	C2		C2	O2		C2	O2	
	C2	H2		O2	HO2		O2	HO2	
	C2	O2		C2	C3		C2	C3	
	O2	HO2		C3	H3		C3	H3	
	C2	C3		C3	O3		C3	O3	

	C3	H3		O3	HO3		O3	HO3	
	C3	O3		C3	C4		C3	C4	
	O3	HO3		C4	H4		C4	H4	
	C3	C4		C4	O4		C4	O4	
	C4	H4		C4	C5		O4	HO4	
	C4	O4		C5	H5		C4	C5	
	C4	C5		C5	C6		C5	H5	
	C5	H5		C6	H61		C5	C6	
	C5	C6		C6	H62		C6	H61	
	C6	H61		C6	O6		C6	H62	
	C6	H62		O6	HO6		C6	O6	
	C6	O6		C5	O5		O6	HO6	
	O6	HO6		O4	+C1		C5	O5	
	C5	O5							
	O4	+C1							
	BL1			GL1			TL1		
No.	Atom	Atomtype	Charge	Atom	Atomtype	Charge	Atom	Atomtype	Charge
0	O4	OC301	-0.36	O4	OC301	-0.36	HO4	HCP1	0.42
1	C4	CC311	0.09	C4	CC311	0.09	O4	OC301	-0.4
2	H4	HCA1	0.09	H4	HCA1	0.09	C4	CC311	0.09
3	C5	CC311	0.11	C5	CC311	0.11	H4	HCA1	0.09
4	C3	CC321	0.05	C3	CC321	0.05	C5	CC311	0.11
5	O5	OC301	-0.4	O5	OC301	-0.4	C3	CC321	0.05
6	H5	HCA1	0.09	H5	HCA1	0.09	O5	OC301	-0.4
7	C6	CC321	0.05	C6	CC321	0.05	H5	HCA1	0.09
8	H3	HCA2	0.09	H3	HCA2	0.09	C6	CC321	0.05
9	O3	OC311	-0.65	O3	OC311	-0.65	H3	HCA2	0.09
10	H31	HCA2	0.09	H31	HCA2	0.09	H31	HCA2	0.09
11	C1	CC311	0.34	C1	CC311	0.29	C1	CC311	0.09
12	H61	HCA2	0.09	H61	HCA2	0.09	H61	HCA2	0.09
13	O6	OC311	-0.65	O6	OC311	-0.65	O6	OC311	-0.65
14	H62	HCA2	0.09	H62	HCA2	0.09	H62	HCA2	0.09
15	HO3	HCP1	0.42	HO3	HCP1	0.42	HO3	HCP1	0.42
16	O1	OC311	-0.65	H1	HCA1	0.09	O3	OC311	-0.65
17	H1	HCA1	0.09	C2	CC321	0.05	H1	HCA1	0.09
18	C2	CC321	0.05	HO6	HCP1	0.42	C2	CC321	0.05
19	HO6	HCP1	0.42	H2	HCA2	0.09	HO6	HCP1	0.42
20	H2	HCA1	0.09	O2	OC311	-0.65	H2	HCA2	0.09
21	O2	OC311	-0.65	H21	HCA2	0.09	O2	OC311	-0.65
22	H21	HCA2	0.09	HO2	HCP1	0.42	H21	HCA2	0.09
23	HO2	HCP1	0.42				HO2	HCP1	0.42
24	HO1	HCP1	0.42						
	Bonds			Bonds			Bonds		
	C1	O1		C1	H1		C1	H1	
	C1	H1		C1	O5		C1	O5	
	O1	HO1		C1	C2		C1	C2	

C1	O5	C2	H2	C2	H2
C1	C2	C2	O2	C2	O2
C2	H2	O2	HO2	O2	HO2
C2	O2	C3	H3	C3	H3
O2	HO2	C3	O3	C3	O3
C3	H3	O3	HO3	O3	HO3
C3	O3	C3	C4	C3	C4
O3	HO3	C4	H4	C4	H4
C3	C4	C4	O4	C4	O4
C4	H4	C4	C5	C4	C5
C4	O4	C5	H5	C5	H5
C4	C5	C5	C6	C5	C6
C5	H5	C6	H61	C6	H61
C5	C6	C6	H62	C6	H62
C6	H61	C6	O6	C6	O6
C6	H62	O6	HO6	O6	HO6
C6	O6	C5	O5	C5	O5
O6	HO6	C3	H31	C3	H31
C5	O5	C2	H21	C2	H21
C3	H31	O4	+C1	O4	HO4
C2	H21				
O4	+C1				

Table 2. Added Definitions to ffbonded.itp in the charmm36-feb2021.ff forcefield.

Angletypes							
i	j	k	func	theta0	ktheta	ub0	kub
CC311	OC301	CC311	5	109.7	794.96	0	0
OC301	CC311	CC311	5	111.5	376.56	0	0
OC301	CC311	CC321	5	111.5	376.56	0	0
HCA1	CC311	OC301	5	109.5	502.08	0	0
CC3161	OC301	CC311	5	109.7	794.96	0	0
HCA1	CC311	CC321	5	110.1	288.696	0.2179	18853.1
HCA1	CC311	CC311	5	110.1	288.696	0.2179	18853.1
CC311	CC311	HCA1	5	110.1	288.696	0.2179	18853.1
OC301	CC311	OC301	5	116.5	376.56	0	0
CC311	OC301	CC3162	5	109.7	794.96	0	0
HCP1	OC301	CC311	5	106	418.4	0	0
OC301	CC311	OC311	5	116.5	376.56	0	0
OC311	CC311	HCA1	5	108.89	460.24	0	0
CC311	CC321	HCA1	5	110.1	288.696	0.2179	18853.1
HCA1	CC321	OC311	5	108.89	460.24	0	0
HCA1	CC321	HCA2	5	109	297.064	0.1802	4518.72
Dihedraltypes							
i	j	k	l	func	phi0	kphi	mult
CC3162	CC3161	OC301	CC311	9	0	0.92048	1

CC3162	CC3161	OC301	CC311	9	180	1.17152	2
CC3162	CC3161	OC301	CC311	9	0	3.72376	3
CC3161	CC3161	OC301	CC311	9	0	0.92048	1
CC3161	CC3161	OC301	CC311	9	180	1.17152	2
CC3161	CC3161	OC301	CC311	9	0	3.72376	3
HCA1	CC3161	OC301	CC311	9	0	1.188256	3
CC3161	OC301	CC311	CC321	9	0	1.6736	1
CC3161	OC301	CC311	CC321	9	0	2.05016	3
CC3161	OC301	CC311	OC301	9	180	0.2092	1
CC3161	OC301	CC311	OC301	9	0	3.80744	2
CC3161	OC301	CC311	OC301	9	180	5.31368	3
CC3161	OC301	CC311	HCA1	9	0	1.188256	3
CC3163	CC3161	OC301	CC311	9	0	0.92048	1
CC3163	CC3161	OC301	CC311	9	180	1.17152	2
CC3163	CC3161	OC301	CC311	9	0	3.72376	3
CC311	OC301	CC311	HCA1	9	0	1.188256	3
CC311	OC301	CC311	CC311	9	0	1.6736	1
CC311	OC301	CC311	CC311	9	0	2.05016	3
CC311	OC301	CC311	CC321	9	0	1.6736	1
CC311	OC301	CC311	CC321	9	0	2.05016	3
CC311	OC301	CC311	OC301	9	180	0.2092	1
CC311	OC301	CC311	OC301	9	0	3.80744	2
CC311	OC301	CC311	OC301	9	180	5.31368	3
OC301	CC311	CC311	OC301	9	0	0.29288	1
OC301	CC311	CC311	OC301	9	180	8.32616	2
OC301	CC311	CC311	OC301	9	180	7.19648	3
OC301	CC311	CC311	OC301	9	180	5.60656	1
OC301	CC311	CC311	OC301	9	0	4.97896	2
OC301	CC311	CC311	OC301	9	0	10.79472	3
HCA1	CC311	CC311	OC301	9	180	0.2092	6
HCA1	CC311	CC311	HCA1	9	0	0.8368	3
HCA1	CC311	CC311	CC321	9	0	0.8368	3
CC321	CC311	CC311	OC301	9	0	0.79496	3
OC301	CC311	CC311	OC311	9	180	11.0876	1
OC301	CC311	CC311	OC311	9	0	0	2
OC301	CC311	CC311	OC311	9	180	0.54392	3
OC301	CC311	CC321	HCA2	9	0	0.8368	3
CC321	CC311	CC311	CC321	9	0	0.8368	3
HCA1	CC311	CC321	HCA2	9	0	0.8368	3
HCA1	CC311	CC311	OC311	9	0	0.58576	3
CC311	CC311	CC311	HCA2	9	0	0.8368	3
CC311	CC311	CC311	OC311	9	0	1.4644	1
CC311	CC311	CC311	OC311	9	0	2.88696	2
CC311	CC311	CC311	OC311	9	180	11.67336	3
HCA1	CC311	CC321	OC311	9	0	0.58576	3
OC301	CC311	CC321	OC311	9	180	11.0876	1

OC301	CC311	CC321	OC311	9	0	0	2
OC301	CC311	CC321	OC311	9	180	0.54392	3
CC311	CC311	CC321	HCA2	9	0	0.8368	3
CC311	CC311	CC321	OC311	9	0	1.4644	1
CC311	CC311	CC321	OC311	9	0	2.88696	2
CC311	CC311	CC321	OC311	9	180	11.67336	3
CC311	CC321	OC311	HCP1	9	0	1.4644	1
CC311	CC321	OC311	HCP1	9	0	1.54808	2
CC311	CC321	OC311	HCP1	9	180	0.79496	3
CC3162	OC301	CC311	HCA1	9	0	1.188256	3
CC3162	OC301	CC311	CC311	9	0	1.6736	1
CC3162	OC301	CC311	CC311	9	0	2.05016	3
CC3162	OC301	CC311	CC321	9	0	1.6736	1
CC3162	OC301	CC311	CC321	9	0	2.05016	3
CC3162	OC301	CC311	OC3C61	9	180	0.2092	1
CC3162	OC301	CC311	OC3C61	9	0	3.80744	2
CC3162	OC301	CC311	OC3C61	9	180	5.31368	3
CC3162	OC301	CC311	CC3161	9	0	1.6736	1
CC3162	OC301	CC311	CC3161	9	0	2.05016	3
CC311	OC301	CC3162	HCA1	9	0	1.188256	3
CC311	OC301	CC3162	OC3C61	9	180	0.2092	1
CC311	OC301	CC3162	OC3C61	9	0	3.80744	2
CC311	OC301	CC3162	OC3C61	9	180	5.31368	3
CC311	OC301	CC3162	CC3161	9	0	1.6736	1
CC311	OC301	CC3162	CC3161	9	0	2.05016	3
HCP1	OC301	CC311	HCA2	9	0	0.75312	3
HCP1	OC301	CC311	OC311	9	0	6.4852	1
HCP1	OC301	CC311	OC311	9	0	4.89528	2
HCP1	OC301	CC311	OC311	9	0	4.47688	3
HCP1	OC301	CC311	HCA1	9	0	0.75312	3
HCP1	OC301	CC311	CC311	9	0	1.00416	3
HCP1	OC301	CC311	CC311	9	0	0.58576	2
HCP1	OC301	CC311	CC311	9	0	4.72792	1
HCP1	OC301	CC311	CC321	9	0	1.00416	3
HCP1	OC301	CC311	CC321	9	0	0.58576	2
HCP1	OC301	CC311	CC321	9	0	4.72792	1
CC311	OC301	CC311	OC311	9	180	1.71544	1
CC311	OC301	CC311	OC311	9	0	3.72376	2
CC311	OC301	CC311	OC311	9	0	0.2092	3
OC301	CC311	OC311	HCP1	9	0	4.89528	1
OC301	CC311	OC311	HCP1	9	0	3.5564	2
OC301	CC311	OC311	HCP1	9	0	1.54808	3
HCA1	CC311	OC311	HCP1	9	0	0.75312	3
CC321	CC311	OC311	HCP1	9	0	1.4644	1
CC321	CC311	OC311	HCP1	9	0	1.54808	2
CC321	CC311	OC311	HCP1	9	180	0.79496	3

OC311	CC311	CC321	HCA1	9	0	0.58576	3
OC311	CC311	CC321	OC311	9	180	11.0876	1
OC311	CC311	CC321	OC311	9	0	0	2
OC311	CC311	CC321	OC311	9	180	0.54392	3
OC311	CC311	CC321	HCA2	9	0	0.8368	3
HCA1	CC321	OC311	HCP1	9	0	0.75312	3
OC301	CC311	CC321	HCA1	9	0	0.58576	3
HCA1	CC311	CC321	HCA1	9	0	0.8368	3

Equilibration was done via a well-tried 21-step method, with an extra step to set the system to the proper temperature for the given simulations, either 296K or 423K (23°C and 150°C).

Table S3. Equilibration scheme for 21+1 step process. Step 22 is to equilibrate the system at the temperature for simulation. The type represents the type of simulation, IE, NVT or NPT. For NPT pressure couplings are: B = Berendsen, PR = Parrinello-Rahman, I = Isotropic.

Step	Time [ns]	Type	Pressure [bar]	Temp [K]
1	0.5	NVT	-	600
2	0.25	NVT	-	300
3	0.5	NPT B I	98.692	300
4	0.25	NVT	-	600
5	0.5	NVT	-	300
6	0.5	NPT B I	2960.77	300
7	0.5	NVT	-	600
8	0.5	NVT	-	300
9	0.5	NPT B I	4934.616	300
10	0.25	NVT	-	600
11	0.5	NVT	-	300
12	0.5	NPT B I	2467.308	300
13	0.25	NVT	-	600
14	0.5	NVT	-	300
15	0.25	NPT B I	493.4616	300
16	0.25	NVT	-	600
17	0.5	NVT	-	300
18	0.5	NPT B I	49.34616	300
19	0.25	NVT	-	600
20	0.5	NVT	-	300
21	20	NPT PR I	1	300
22	10	NPT PR I	1	293/423

Tensile simulations was performed using Parrinello-Rahman semiisotropic pressure coupling for 6/7 ns, until the initial box length was stretched over 100%. It was deformed at a rate of 0.001 nm/ps in one direction (X).
